# Inferring modes of evolution from colorectal cancer with residual polyp of origin

**DOI:** 10.18632/oncotarget.23687

**Published:** 2017-12-26

**Authors:** Minsoo Kim, Brooke R. Druliner, Nikolaos Vasmatzis, Taejeong Bae, Nicholas Chia, Alexej Abyzov, Lisa A. Boardman

**Affiliations:** ^1^ Program in Bioinformatics and Computational Biology, University of Minnesota, Minneapolis, MN 55455, USA; ^2^ Department of Health Sciences Research, Center for Individualized Medicine, Mayo Clinic, Rochester, MN 55905, USA; ^3^ Division of Gastroenterology and Hepatology, Mayo Clinic, Rochester, MN 55905, USA

**Keywords:** colorectal cancer, adenomatous polyp, cancer evolution, carcinogenesis, mutation

## Abstract

Besides the classical evolutionary model of colorectal cancer (CRC) defined by the stepwise accumulation of mutations in which normal epithelium transforms through an intermediary polyp stage to cancer, a few studies have proposed alternative modes of evolution (MOE): early eruptive subclonal expansion, branching of the subclones in parallel evolution, and neutral evolution. However, frequencies of MOEs and their connection to mutational characteristics of cancer remain elusive. In this study, we analyzed patterns of somatic single nucleotide variations (SNVs) and copy number aberrations (CNAs) in CRC with residual polyp of origin from 13 patients in order to determine this relationship. For each MOE we defined an expected pattern with characteristic features of allele frequency distributions for SNVs in cancers and their matching adenomas. From these distinct patterns, we then assigned an MOE to each CRC case and found that stepwise progression was the most common (70% of cases). We found that CRC with the same MOE may exhibit different mutational spectra, suggesting that different mutational mechanisms can result in the same MOE. Inversely, cancers with different MOEs can have the same mutational spectrum, suggesting that the same mutational mechanism can lead to different MOEs. The types of somatic substitutions, distribution of CNAs across genome, and mutated pathways did not correlate with MOEs. As this could be due to small sample size, these relations warrant further investigation. Our study paves the way to connect MOE with clinical and mutational characteristics not only in CRC but also to neoplastic transformation in other cancers.

## INTRODUCTION

The foundation for the studies of genetic evolution in many cancer types was built upon the finding first presented in the seminal work by Fearon and Vogelstein that the accumulation of genetic alterations led to neoplastic transformation in the colon to colorectal cancer (CRC) [1]. This widely accepted and dominant paradigm that CRC arises in a linear model of accumulated genetic mutation and large-scale genomic disruptions of chromosomal instability continues to be the infrastructure upon which extensive research on carcinogenesis is based [2]. In this classical model of CRC, carcinogenesis is presumed to follow a linear trajectory from normal colon tissue to a precancerous lesion, known as an adenomatous polyp, to cancer.

Since the introduction of next generation sequencing technology, improved ability to accurately characterize cancer genomes allowed researchers to explore and challenge this idea of sequential progression in carcinogenesis of CRC and this effort resulted in three additional evolutionary models to address carcinogenesis. One recent study on the distribution of somatic mutations in CRC proposed an extension of the linear model into a quick eruptive accumulation of mutations in the polyp followed by subclonal competition and a plateau of extensive mutation accumulation in the resulting cancer [3]. This phenomenon results from a lack of selection pressure in combination with high rate of mutation accumulation. One noteworthy aspect of this model, also known as the ‘Big Bang,’ is that it describes how early mutations shape the high intratumoral heterogeneity (ITH) observed in the late stage of CRC and may be associated with a more aggressive clinical behavior and decreased rates of survival [4].

Another study postulated a parallel evolution involving a separate lineage of private, cancer- and adenoma-specific mutations branching out from the early clonal mutations shared between the two tissues [5]. Due to this divergence, cancer exhibits a different mutational architecture than the traditionally expected expansion of the subclonal populations present in the polyp. In parallel evolution, driver mutations may be present in subclones and multiple independent subclonal expansions may persist so long as they are of equal fitness. Thus one subclone does not confer a selective advantage over another subclone, which may in fact have the same driver mutation but unique private mutations. In some cases the polyp accumulates a greater number of mutations than cancer, while in others the cancer accumulates more mutations than the polyp, suggesting that the number of mutations alone cannot determine whether a CRC will undergo parallel evolution.

Lastly, the possibility of cancer evolution following a simple neutral power-law was explored based on the finding that some cancers exhibit several distinct distributions of the allele frequency of somatic mutations in their cancer lineage in which one evinces selection pressure while another does not. The notion is that the distribution of passenger mutations with low allele frequencies with respect to the clonal mutations near the allele frequency of 0.5 would follow a 1/f distribution [6]. On the other hand, a lineage exerting selection pressure would have driver mutations represented as an additional subclonal peak located between the peak of the passenger mutations and that of the clonal mutations [7].

These four Modes of Evolution (MOEs), stepwise, eruptive, parallel, and neutral, offer possible explanations for the temporal relationship among the different types of genome wide alterations as well as intra- and inter-tumoral heterogeneity. The importance of knowing the features of MOEs with the highest impact on the polyp or tumor's behavior is highlighted by the genetic evolution in glioblastoma multiforme (GBM). In one study, the degree of persistent mutations from the primary tumor that were also present in the recurrent tumor differed based on the MOE of the primary tumor [8]. The authors found that the recurrent tumors carried 75% of the mutations present in the primary tumor with linear MOE compared to those with parallel MOE in which recurrent tumors shared only 25% of the mutations in the primary tumor. The recurrence of the cancer, which rarely can be successfully treated and cured, was genetically represented by those early mutations present in the primary tumor that had developed resistance to chemotherapy. If it were possible to recognize all functionally relevant mutations in the primary tumor and to develop treatments for each of these mutations, conceivably it would be possible to prevent recurrences. Thus, studying the MOEs and understanding the characteristics of clinical cases in relation to the evolutionary path that led to malignant transformation may be leveraged to improve accurate prognostication and provide targets for personalized treatment options.

**Figure 1:**
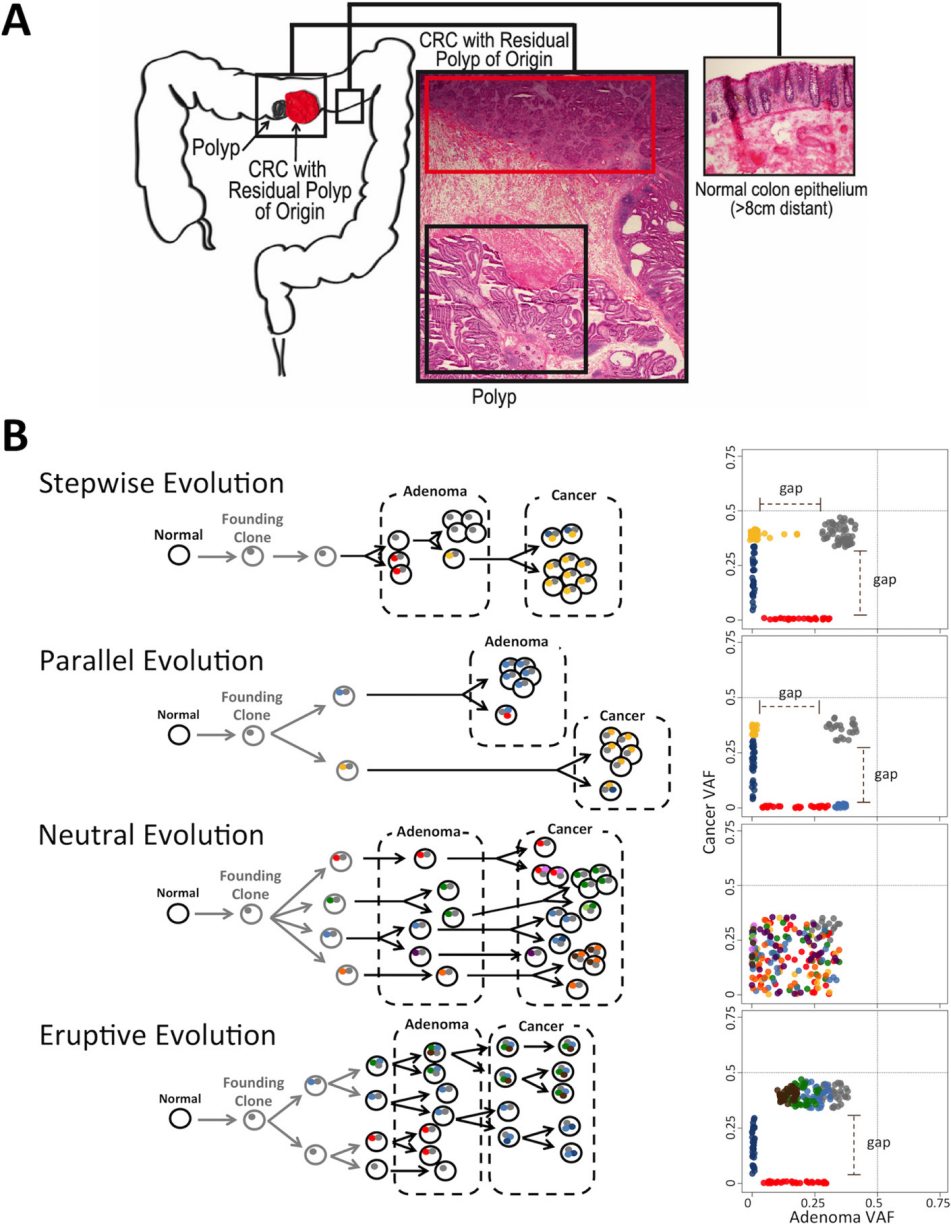
Modes of Evolution (MOE) in the transformation from adenoma to cancer and resulting AF distributions (**A**) CRC with residual polyp of origin (top red box) refers to the cancer that has the polyp located physically adjacent to the cancer (bottom black box). (**B**) Schematic representation of MOEs in adenoma-to-cancer transformation: classical sequential stepwise evolution, parallel (branched) model, neutral evolution, and eruptive (Big-Bang) model of early bursts of single or whole chromosomal genomic disruption. Circles and dots depict cells and mutations respectively. Colors correspond to different clones/subclones. Plots on the right represent 2D AF distributions of somatic variants in polyps and cancer with dotted lines on 0.5 AF. AFs of shared SNVs could be shifted from 0.5 due to sample impurity. These distributions are characteristic of each mode and can be used for MOE prediction.

We previously reported that at least 10% of CRC have the contiguous residual polyp of origin (CRC RPO+) identifiable in the surgically resected specimen [9]. We performed whole genome sequencing and analysis of such CRC RPO+ cases, which included matched peripheral blood leukocytes, normal colon a minimum of 8 cm distant from the polyp and/or cancer edge, the cancer adjacent polyp (CAP) and the contiguous CRC (Figure 1A). Comparative analysis between CRC RPO+ and CRC without residual polyp of origin (RPO-) revealed essentially the same histology, gene expression patterns, mutated genes/pathways, as well as the same stage-adjusted disease free and overall survival. Similarly, the CAP component is highly likely to represent the intermediary state between normal colon epithelium and CRC RPO+ because it is in direct contiguity with the cancer. This strongly argues that CRC RPO+ is a valid model to study neoplastic transformation in the colon [9].

In this study, we analyzed patterns of somatic mutations in the CRC RPO+ cases to determine the relationship between different MOEs in the transformation from normal colon cells to CRC and mutational characteristics of CRC RPO+ cases. We used the same samples from our previous study but excluded those without a matched normal colon epithelial tissue for a total of 13 cases (Supplementary Table 1). Four of these cases were clinically determined to be aggressive, having either recurred or presented as advanced stage IV disease.

## RESULTS

### Utility of mutation AFs across neoplastic transformation for MOE classification

We observed distinct patterns in the allele frequency distribution of each pair of cancer and matching polyp characterized by 1) two gaps in AF; 2) one gap in AF; or 3) no gap (Supplementary Figures 1–11). We hypothesized that these patterns are representative of CRC evolution. Consequently, we derived an expected pattern of AF distributions in CRC and its corresponding polyp for each MOE based on its key characteristics (Figure 1B). For stepwise and eruptive MOEs, it is expected that most SNVs are shared between the adenoma and its corresponding cancer. Moreover, selective pressure, which increases the AF of certain subclonal mutations nearly to the level of AF present in shared clonal mutations, is a common feature in stepwise MOE. For parallel MOE, where the adenoma and cancer branch early in their evolution to independent pathways, most SNVs are expected to accumulate after the branch point between a polyp and cancer. Thus, most of these SNVs are polyp or cancer specific, rather than shared. One important distinction between stepwise and parallel MOEs is that the parallel MOE has adenoma and cancer separately evolving along their respective subclonal lineage, implying distinct adenoma- and cancer-specific mutations conferring a growth advantage for each tissue compartment. Thus, the AF of private mutations in polyp compartment of parallel MOE cases would be close to 0.5 (blue dots in Figure 1B) while for stepwise MOE this is not the case. A key feature of neutral MOE is the little to no selective pressure represented by a lack of clear distinction between early-shared (gray cluster) and late-shared mutations (the rest). CRC originating via an eruptive MOE is characterized by the development of all shared clonal mutations prior to the pre-cancer polyp phase followed by little to no selection so that the shared clonal mutations early in transformation would include clusters of early mutations (brown, green, and light blue cluster) in addition to the grey cluster of SNVs.

The anticipated AF distribution for the union of somatic SNVs per patient (i.e., discovered both in adenoma and cancer) is a characteristic feature of MOE in both the adenoma and cancer compartments. Gradual transition from adenoma to cancer in stepwise MOE will result in a gap in the AF distribution in cancer and small or no gap in adenoma. However, no such gaps are expected in the distributions for neutral MOE, as SNVs with all AFs are shared between adenoma and cancer. Long independent evolution of adenoma and cancer in parallel MOE will result in clear gaps in the two distributions. Early shaping of the shared clonal mutations in eruptive MOE will result in a gap in AF distribution of adenoma, but at no or less pronounced gap in AF distribution of cancer.

Purity of a sample, i.e., fraction of malignant cells, is generally determined by a pathologist visually inspecting histological slides of the tissue and the purity level varies from sample to sample [10]. Lower purity level in adenoma sample would shift down the overall somatic SNV AF distribution in adenoma towards zero and could potentially decrease the gap observable in the 2D plot. However, while purity level can shift, shrink, or expand the AF distribution and affect the absolute AF, the relative pattern of the distribution as a whole remains the same. Thus, comparison of the relative AF between the shared and the private mutations can be used to infer the existence of selective pressure in a CRC without the influence of the purity level difference across samples.

CRCs also exhibit large chromosomal aneuploidies with deletion or duplication of the entire chromosomes and/or chromosomal arms, which can be characterized in a MOE-specific manner. Aneuploidies are expected to be noticeable beginning in adenoma for eruptive MOEs, in which most of the copy number alterations happen early in the lineage and the cancer grows out of a clone present in the polyp stage. In an independent evolution of polyp and CRC, as in parallel MOE, aneuploidies should only be observed in cancer. Similarly, in the stepwise evolution, deleterious early copy number alterations would be selected out, leaving only the chromosomally stable clone in polyp stage to grow into cancer, at which the growth could tolerate larger scale chromosomal changes. The neutral MOE's copy number alterations status can be more difficult to interpret because the lack of selection could imply a possibility of smaller copy number alterations but never a larger, more damaging copy number alteration that only cells with selective advantage could tolerate.

Here, it is crucial to note that aneuploidies would not affect the shape of the distribution in the 2D plot for several reasons. First, large regions of the genome are chromosomally stable in most of the polyp cases. In fact, only three out of the 13 CAP cases have greater than 10% of its genome affected by aneuploidies. Another reason is that even if the adenoma is affected by aneuploidies, they are mostly subclonal and will not shift the AF of the private mutations significantly. This, along with the simultaneous shift in AF between the shared and the private SNVs mentioned above, support the notion that aneuploidies are unlikely to affect our interpretation of the 2D plot in classifying cases by MOEs. All of these reasons also apply to genome doubling. Genome doubling, which is found to be commonly associated with increased rate of evolutionary growth in colorectal cancer, occurs early in the development and can either affect both adenoma and cancer to raise the AF distributions in both axes or, again, affect the entire tissue so that the AF in both the private and shared mutations shift together. Therefore, using the relative position of the AF in private mutations with respect to the shared mutations serves as a strong criterion in determining the MOE of a case.

### Comparative example of stepwise and eruptive MOEs

**Figure 2:**
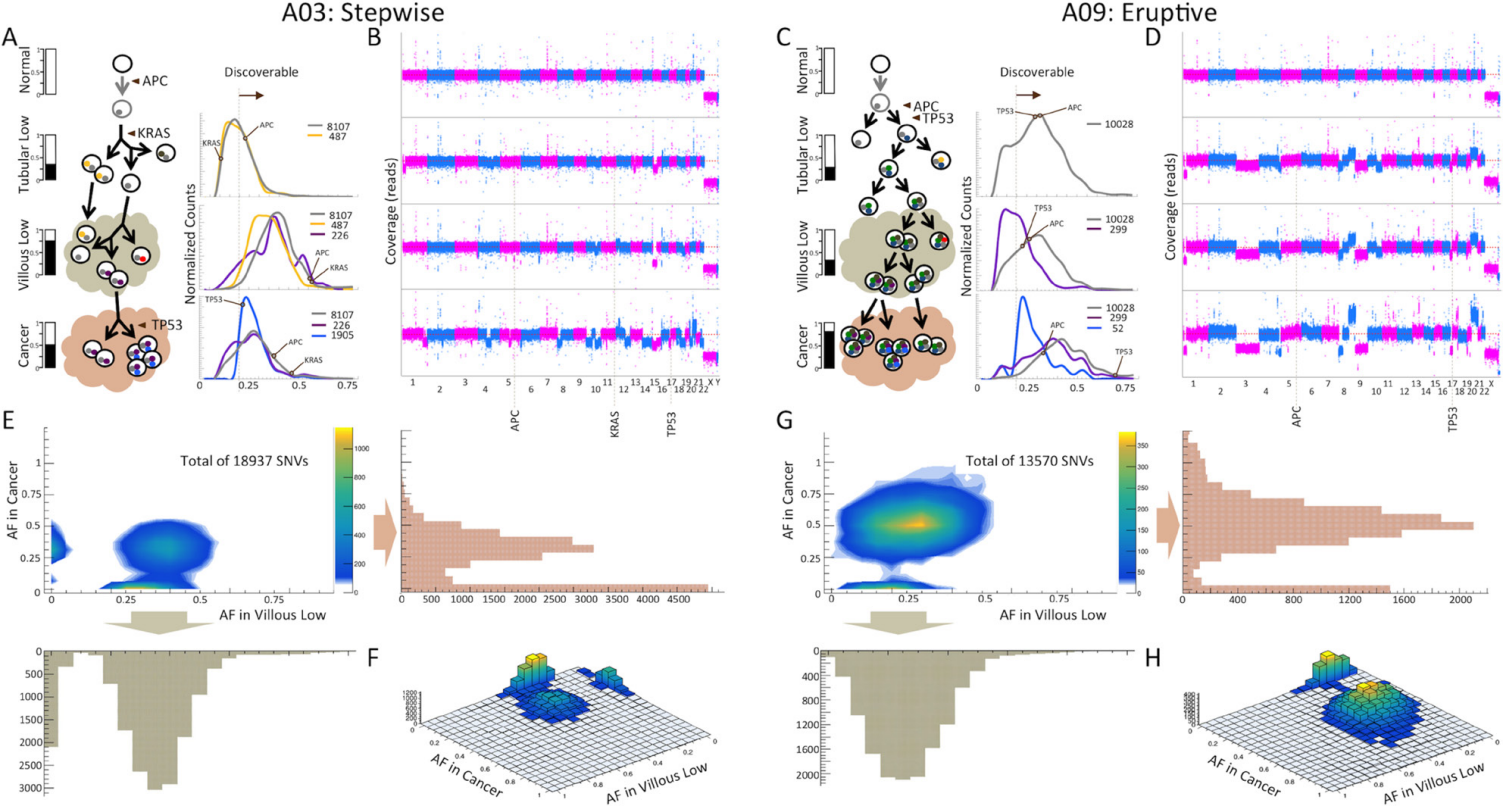
Example of a stepwise (A03) and an eruptive MOE (A09) revealed by somatic mutations analysis (**A** and **C**) Schematic representation of models for origin, presence, and propagation of clonal and subclonal mutations at each stage of transformation. Mutations in *APC*, *KRAS,* and *TP53* are labeled at the corresponding stages of the evolution. The bars on the left represent estimated sample purity from SNV AF and CNA analyses. Distributions on the right represent the mutations shared by adenomas and cancer (in gray), those shared between the villous low and cancer (in purple), and those that are cancer-specific (in blue). Vertical dashed lines represent an approximate detection limit with our analytical pipeline. SNVs with AFs below detection limit are discovered in other samples. The middle shows schematics of presumed clonal lineage evolution. (**B** and **D**) Genome copy number profiles at each stage. In case A03, large aneuploidies are observed only in the cancer stage. In case A09, large aneuploidies are observed in the tubular stage and are maintained until the cancer stage. (**E** and **G**) Distributions of AF for somatic SNVs are consistent with stepwise MOE in A03 and eruptive MOE in A09 (Figure 1B). (**F** and **H**) 3D representation of the AF distributions of SNVs is shown for each case. The height of the distributions, which shows the number of mutations, indicates large fraction of both shared SNVs and private SNVs for A03 and large fraction of polyp-specific SNVs and shared SNVs for A09.

Let's consider two cases of neoplastic transformation to CRC in our cohort: in case A03, where we classified MOE as stepwise, and in case A09, where we classified MOE as eruptive (Figure 2). In each patient, two stages of adenoma (tubular and villous) were observed, harvested, and sequenced. In these cases, the tubular and villous polyp along with the corresponding resultant cancer were in direct contiguity and present on the same histology slide, which likely represents the tissue compartments involved in the malignancy that patient A03 developed. Tubular adenoma in A03 had 5,611 somatic SNVs with no aneuploidies. Tubular adenoma in A09 had 8,786 somatic SNVs with apparent aneuploidies. As expected from the classical Fearon and Vogelstein model, both the tubular and villous polyps had a stop mutation in *APC*, which is a gene recognized to be mutated in many CRC and considered to be involved in initiating neoplastic transformation in the colon. Each of the patients also had mutations in one of two well-known cancer-driver genes: in *KRAS* and *TP53* in the patient case A03 and in *TP53* in the patient case A09. The introduction of the *TP53* also correlated with observation of large amounts of aneuploidies (Figure 2B, 2D). In both patient cases, evolution to villous adenoma did not change the copy number profile though the copy number alterations became more pronounced, likely as a result of better sample purity in the villous compared to the corresponding tubular polyp compartment. This is particularly noticeable in case A03, in which more somatic SNVs are detected in the villous polyp than in its corresponding tubular adenoma, 14,393 vs. 5,611. Consistently, AF of early mutations originated in tubular, including stop mutations in *APC* and *KRAS,* are increasing and are centered close to 50%, suggesting that one clone dominates this stage. In A09, count of SNVs increase only slightly, 8,893 vs. 8,786, with AF of early mutations, including stop mutations in *APC* and *TP53,* unchanged. As an average AF of early mutations is significantly below 50%, mutation-containing cells likely constitute only a small fraction of all cells in the villous polyp.

The copy number profile in the cancer from patient A09 is the same as, but much more pronounced, in the villous tissues, suggesting higher purity of cancer cells in the cancer compartment than in the villous tissues. In agreement with this, more somatic SNVs are detected in cancer compared to the villous polyp, 11,357 vs. 8,893, and the AF of early mutations in the adenoma are centered close to 50%, suggesting high purity level in this sample. Deletion of the other (not mutated) copy of *TP53* gene in the villous compartment leads to an even higher AF of a stop mutation in this gene. New mutations only found in cancer have much lower AF, indicating that these mutations represent subclones in the cancer. Large aneuploidies becoming progressively more definitive since their introduction early in the lineage is consistent with eruptive progression.

Contrary to this, aneuploidies and CNAs in A03 are present only in cancer, in which a stop mutation is observed in *TP53*. However, number of detectable somatic SNVs decreases as compared to villous, 8,831 vs. 14,393, with AF of mutation observed in adenoma also decreasing. This is most likely due to lower purity in the cancer component. New mutations found only in cancer are centered at a similar AF despite being subclonal, suggesting a significant selective advantage. All these observations are consistent with the gradual accumulation of mutations followed by a selective pressure that progressively alters the genomic landscape mostly with SNVs until the late stage of cancer, at which point there are large aneuploidies and CNAs.

### Rules for classifying MOEs

We defined a set of rules suggesting MOE based on each characteristic signature that can be observed in spatial-temporal pattern of SNVs (Table 1). Five rules were defined to describe characteristics of each MOE. These rules were based on the theoretical expectations for the four considered MOEs (Figure 1B) and are related to the number of SNVs, the shape of the SNV AF distribution in adenoma and cancer, and the progression of CNAs.

**Table 1:** Rules to classify MOE for each case of neoplastic transformation

Rules/MOE	Stepwise	Parallel	Neutral	Eruptive
#1: Fraction of private SNVs relative to shared SNVs	Large fraction of private in cancer and in polyp as well as shared mutations (qualitatively: ≥10% cancer specific, ≥10% cancer specific, and ≥40% shared SNVs)	Majority is private in cancer and in polyp (qualitatively: ≥50% either cancer or polyp specific SNVs)	Majority is shared (qualitatively: <5% cancer specific and polyp specific SNVs)	Majority is private in polyp and shared (qualitatively: <5% cancer specific, ≥10% polyp specific, and ≥80% shared SNVs)
#2: Gap in the SNV AF distribution in polyp and in cancer	Yes/Yes	Yes/Yes	No/No	No/Yes
#3: Position of private SNV clusters relative to the shared SNV cluster	Polyp-specific SNV AF is lower than the shared SNV AF	Both polyp and cancer-specific SNV AF are equal to the shared SNV AF	No private mutations	Both polyp and cancer-specific SNV AFs are lower than the shared SNV AF
#4: Indistinguishable early shared from later shared SNVs	No	No	Yes	Yes
#5: Numerous aneuploidies start in	Cancer (Late)	Cancer (Late)	N/A	Polyp (Early)

Rule #1 is comparing the number of adenoma- and cancer-specific somatic SNVs with the number of somatic SNVs shared between them. Rule #2 is testing whether a gap exists in the AF distribution of somatic SNVs in a 2D plot of adenoma vs. cancer (Figure 1B). Since selection pressure within a lineage would shift the private SNV AFs further away from the shared SNV AFs, a larger gap would signify mutational architecture in later stages that is far different from its shared, early clonal mutations. Rule #3 compares the spatial distribution of the shared SNV AFs with respect to the private SNV AFs. In evolutionary scenarios with selective pressure, almost all of the later cell population would derive from a cell that acquired driver mutations early in the lineage. The high prevalence of this cell leads to a private SNV AF that nearly matches the SNV AF of the shared, clonal SNV AF. Rule #4 examines whether the early shared SNVs and the later shared SNVs are distinguishable based on AF distribution. Although similar to rule #2, this rule requires both adenoma- and cancer-specific mutations, which will filter out neutral evolution in which the lack of selection pressure leads to all the mutations in the adenoma to be carried forward to the cancer. Similarly, this rule filters out eruptive evolution given that in eruptive evolution the majority of the mutations found in the cancer similar to those in the adenoma due to the late steady state in which mutations infrequently accumulate in the CRC beyond those that rapidly accumulated in the early precancerous polyp phase and persisted into the cancer. Lastly, rule #5 compares the earliest time point at which large aneuploidies are noticeable.

We then applied majority vote from all rules to classify MOE for each analyzed CRC case (Table 2, Supplementary Figures 1–11). We found that approximately 70% of CRCs in our dataset evolved in a stepwise or a parallel MOE. One case demonstrated an eruptive MOE and three cases neutral MOE. Cases A11 and A13 both had a gap in the SNV AF distribution in the adenoma but not in the cancer, which corresponded to none of the characteristics of MOEs outlined earlier. For A13, the majority of the rules did not apply because of the lack of distinction between the private and the shared SNV AF distribution in addition to the unusual characteristics in the SNV AF gap between the adenoma and its related cancer. One explanation for the unusual gap in the 2D plot is a low purity level in the cancer compared to the adenoma, which is supported by the overall low AF distribution in cancer near 0.25. However, if that were indeed the case, it would still not explain the fact that the private mutations in cancer appear to have a higher AF than the shared SNVs. In fact, pathology review indicates that the macrodissected portion of the cancer had 60% tumor density (Supplementary Table 2). For these reasons, cancer-specific mutations shifting relative to the mutations shared between both adenoma and cancer can be attributed to another possible explanation that the CRC and the physically adjacent CAP for cases A11 and A13 arose from different clones independently rather than the CRC growing from the same clone as the CAP. This is supported by the AFs of the shared driver mutations *TP53* and *KRAS*. For A11, mutations on *TP53* have AFs of 0.471 and 0.324 in villous adenoma that are decreased to 0.167 and 0.152 in cancer. Similarly, A13 has a mutation on *TP53* with AF of 0.424 in villous adenoma that is decreased to 0.028 in cancer and a mutation on *KRAS* with 0.261 AF only observed in villous adenoma. Lastly, there is also a possibility that these cases represent an additional MOE of extremely rare polyclonal CRC [11]. Thus, we only applied the rules #1 and #5 that were pertinent to the case A13 and this limitation led to a classification that is less reliable than other cases but the relevant rule-based characteristics strongly suggested parallel MOE.

**Table 2:** Rule-based classification of each neoplastic transformation case

Cases/Rules	Rule #1	Rule #2	Rule #3	Rule #4	Rule #5	Conclusion
A02	s	s,p	p	s,p	s,p,n	s,p
A03	s	s,p	s	s,p	s,p,n	s
A04	n	n	n	n,e	n,e	n
A07	s	s,p	p	s,p	s,p,n	s,p
A08	p	s,p	p	s,p	s,p,n	p
A09	e	e	s,e	n,e	n,e	e
A10	n	n	n	n,e	-	n
A11	s	-	s	s,p	n,e	s
A12	s	s,p	p	s,p	-	s,p
A13	p	-	-	-	n,e or s,p,n	Most likely p
A14	n	n	n	n,e	-	n
A15	e	s,p	p	s,p	-	p
A16	s	s,p	s	s,p	-	s

The label s stands for stepwise, p stands for parallel, n stands for neutral, e stands for eruptive, and s,p for a combination of stepwise and parallel. Based on its characteristics relevant to the rule in question, the samples were given the most likely MOE assignments for each rule. Then, each sample was assigned the final MOE with the majority count across the five rules.

It should be noted here that due to the nature of synchronous residual polyp of origin, all patient cases exhibit the property of branching evolution to some extent, even though it is assumed that the cancer sample developed from the adenoma. This is the reason we labeled a few cases to have an MOE of s,p as we could not conclusively classify these cases as having stepwise vs. parallel MOE. Also, the striking feature of neutral MOE is its lack of selective pressure in its lineage, which allows aneuploidies to begin at any point in time as a result of random drift of a clone with aneuploidies to noticeable high frequency. All samples with observable aneuploidies were given the additional neutral MOE assignment for rule #5 because of this inability to predict the initiation of aneuploidies. Lastly, samples without significant aneuploidies were not considered for rule #5.

### Mutational spectra and MOEs

**Figure 3:**
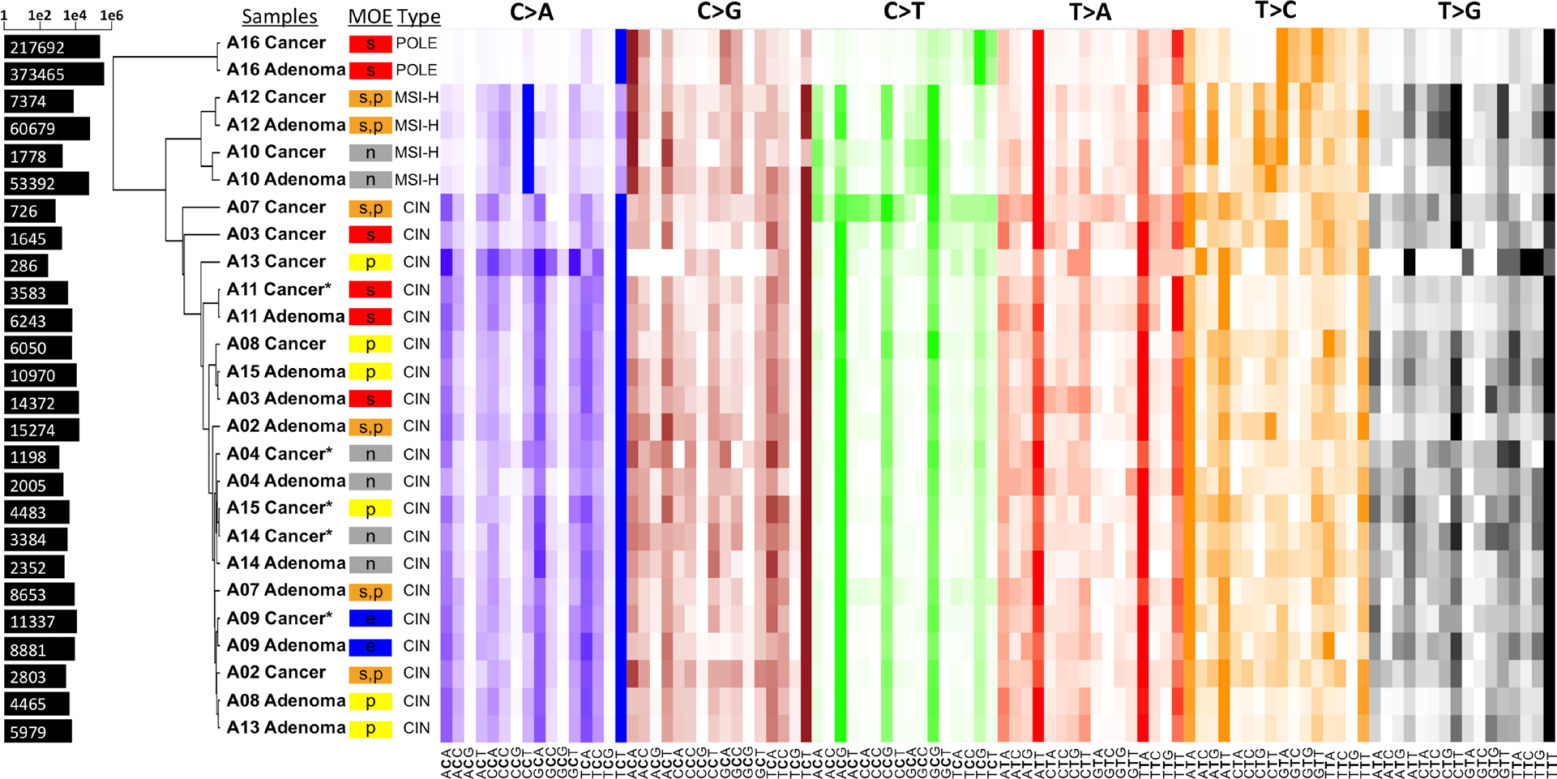
Heatmap of the mutational spectra analysis and hierarchical clustering of all cases Each colored panel represent the 16 possible tri-nucleotide combinations corresponding to the respective transversion or transition type. The color intensity indicates the proportion of the particular tri-nucleotide for that substitution mutation. Bars on the left represent the total number of SNVs for each sample in log scale. Clearly visible linear pattern over all the samples with high color intensity suggests mutational components that are similar in the majority of cases. The samples are clustered into three major clusters with A16 samples being in one, A12 and A10 in another, and the rest in one cluster. A few samples, indicated by the asterisk, had too few cancer-specific mutations. For these samples, the somatic mutations included those found in the villous low adenoma stage. Stepwise MOEs are shown in red, parallel in yellow, the combination of two in orange, neutral in gray, and eruptive in blue.

Next, we broke down the somatic mutations into 96 tri-nucleotide motifs for each sample, as Alexandrov previously did in cancer in order to decipher mutational signatures [12]. Then, we performed a pairwise comparison, and ordered them by hierarchical clustering in order to identify any mutational patterns specific to different MOEs (Figure 3). C>T transitions and C>A transversions seem to contribute the most in distinguishing the clusters apart. Additionally, the similarity in the mutational patterns of cancer and their matched adenoma samples imply that the mechanism resulting in the shaping of the mutational spectra is determined even before adenoma and remains relatively stable in the corresponding cancer.

Clustering of the CAP and cancer samples based on their somatic mutational spectra clearly show three major groups of the samples with the same subtypes: case A16 being in one, cases A10 and A12 being in another, and the rest being in another group. In other words, the subtyping into microsatellite-high (MSI-H), which are defecting for mismatch repair, and microsatellite stable (MSS) cases contributes more significantly to the substitution mutation patterns than the MOE assignment does. Nevertheless, the same subtype does not guarantee the same MOE assignment and this is consistent with two microsatellite-high (MSI-H) cases of A10 and A12 having a different MOE. Although the same underlying genetic hypermutability results in the two cases having a similar number of mutations, A10 cancer had a neutral MOE while A12 cancer underwent a stepwise or parallel MOE. The genetic hypermutability in both A10 and A12 cases were confirmed in their mutational patterns that are similar to signature 6. According to Alexandrov, signature 6 is commonly observed in CRC and is presumed to be associated with compromised DNA mismatch repair (Supplementary Figures 12 and 13). Additionally, the cluster analysis illustrates that the MOE classification is not solely dependent on the number of mutations or the MSI status. Despite the A16 cancer sample having approximately 62 times the amount of somatic mutations found in MSS cases and 6.7 times the amount found in MSI-H cases, it is classified as stepwise MOE as are most of the other cases. The rest of the samples, representing the MSS cases, exhibited distinct patterns of signature 1, which is presumed to be associated with sporadic deamination of 5-methylcytosine. These cases also exhibited spectra similar to signature 5, but signature 5 is not yet known to be associated with particular mechanisms (Supplementary Figure 14).

Having such a high mutation burden, the case A16 was presumed to have mutations in *POLE*, because the adenoma and cancer mutational spectra matched signature 10 in the COSMIC database (Supplementary Figure 15). Signature 10 is commonly observed in CRC and is thought to be associated with variants in DNA polymerase epsilon (*POLE*), which suggests a defect in the *POLE* gene [12]. A missense somatic mutation call in *POLE* with an AF of 0.25 in the cancer sample of the A16 case may account for only a part of the story, since the mutational spectra tell us that the mutations in POLE gene happened early in the evolution for such patterns to form both in adenoma and in cancer. Indeed, we found two missense mutations in the matched normal sample of the case A16. While these could be either germline mutations or somatic mutations that occurred early in development, these mutations could possibly explain the ultra-high mutation rate observed in both the polyp and cancer. The first missense mutation is a known SNP on chromosome 12 at position 133220526 that is predicted to have a slight deleterious effect with a SIFT score of 0.03, which is contrary to a benign effect predicted by the PolyPhen2 score of 0.104 (SNP ID rs5744934). The second missense mutation, which we believe to be responsible for the ultra-high mutation effect, is a variant that has not been previously reported and is located on chromosome 12 at position 133220556. Both SIFT and PolyPhen2 predict the resultant amino acid change from arginine to proline to have a damaging effect with scores of 0.01 and 0.997, respectively. These results imply that defects in specific pathways occurring early in the lineage might contribute more to the shaping of these mutational spectra than the particular MOE that led to the tumor, though the relationship of the ultra-high mutation rate and the MOE requires further study in a larger sample set.

### Aneuploidies and MOE

**Figure 4:**
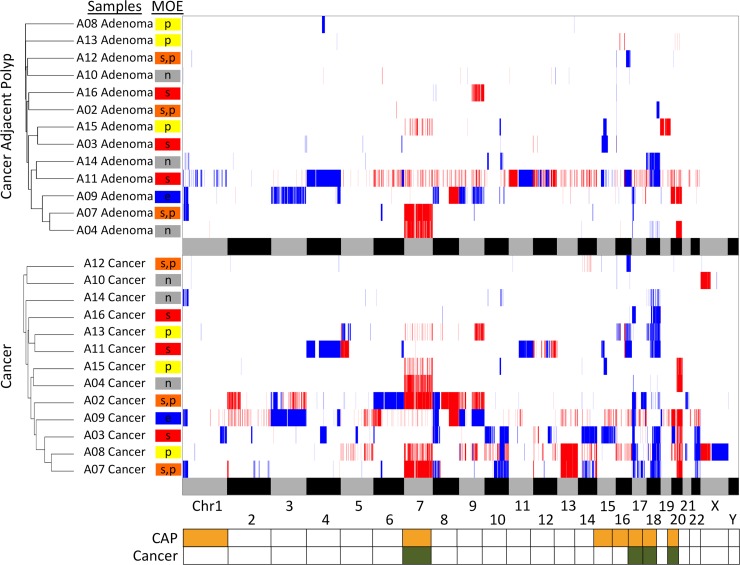
Heatmap of the CNA analysis and hierarchical clustering of the cases by adenoma and carcinoma CNAs in each sample are indicated by either deletions (blue) or duplications (red). The alternating grey and black bars at the bottom of each panel represent the spanning of the chromosomes for regional reference. Samples are grouped together by similarity in pairwise CNA comparison using UPGMA and are labeled with the corresponding MOE. The bottom panel is the summary of chromosomes with the most recurrent changes. Chromosomes with significantly more recurrent changes in CAP are represented by the orange yellow color, and cancer by the olive green color. The stepwise MOEs are shown in red, parallel in yellow, the combination of two in orange, neutral in gray, and eruptive in blue.

To determine if a pattern in the CNAs have specific connections to the MOEs, we devised a pairwise similarity metric characterizing a chromosomal region of duplications or deletions present in both of the samples. The scoring emphasizes only the similarity in the pairs and thus gives a small regional variation the same weight as an entire chromosomal aberration as long as they are present in both samples. A chromosome can have a score between 0 and 1, and the score is higher if more samples have overlapping regions of copy number aberrations for this particular chromosome compared to the other chromosomes. Because these are scores per chromosome, calculating the summation of these scores represents a similarity score between a pair of samples across their entire genome. Samples corresponding to each CAP and cancer tissue types were separately compared and clustered based on these values (Figure 4).

In addition to comparing the CNAs across the samples, this similarity metric also allowed per-chromosome analysis to identify chromosomes with more recurrent CNAs compared to other chromosomes for each CAP and cancer tissue type. Because we often observe higher aneuploidy level in cancer as opposed to adenoma, a comparison between the two is not meaningful. Nevertheless, identification of potential markers is an essential step towards understanding the clinical significance of polyp compared to cancer tissues in addition to the MOE classification. Chromosomes 7, 17, 20, and 18 had the most recurrent copy number alterations across cancer cases, while chromosomes 1, 16, 17, 18, 15, 20, and 7 were most recurrent across CAP cases (Supplementary Figure 16). Duplications in chromosome 7 are significantly recurrent in both CAP (*p*-value of 4.8 × 10^−7^) and cancer (*p*-value of 4.2 × 10^−5^). Deletions in chromosomes 17 and 18 are also significantly recurrent compared to other chromosomes in both CAP and cancer (*p*-values < 3.4 × 10^−5^).

### Utility of exome sequencing and low-coverage in MOE classification

To determine if our criteria for MOE classification is also applicable to exome sequencing data, the 2D plot for case A03 and A09 were re-created based on AF of the somatic SNVs found in coding regions only (Supplementary Figure 17). All features of the 2D plot from the whole genome sequencing data were observable in the new 2D plot. Those cases with fewer number of SNVs may lose some features of the AF distribution simply due to the lower number of SNVs in the exome compared to the genome, but the criteria appears to be robust to exome sequencing overall. Similarly, the differential occurrence of aneuploidies may still be observable despite the less definitive amplification and CNAs from the exome. Our criteria should be applied to an actual exome sequencing data to determine whether the MOE classification can still apply to exome sequencing. We downsampled our whole genome sequencing data to 10 × coverage in order to determine if the MOE classification is possible with low-coverage data. Despite relaxing the parameters to include SNVs called by at least two callers, instead of three, only a limited number of shared mutations could be detected. As precision of AF estimates also reduced, no confident MOE classification would be made (data not shown). Together, these findings illustrate that a higher sequencing depth is more essential than the fraction of analyzed genome for MOE classification.

## DISCUSSION

There have been several studies modeling the dynamic process of neoplastic transformation based on the spatial characterization of cancer by multi-region sampling as well as temporal progression of cancer by comparing the primary cancer sample to metastases [3, 13]. Traditionally intra-tumor heterogeneity from multiple spatially distinct regions of a cancer at one point in time or possibly in corresponding cancer recurrence tissues has been one means to evaluate the clonal history of the tumor and create phylogenetic trees that depict cancer evolution [14, 15]. However, these studies do not take into account information of intermediate clones that do not persist through malignant transformation. Even if they do, they are often exclusively based on unrelated polyps that have not developed into cancer or cancers in which the presumed polyp of origin is no longer present, i.e., without directly evaluating the molecular transformation of normal colon through polyp to cancer in the same person. In addition to the studies on spatial characterization of cancer, single cell sequencing studies have contributed in the in-depth analyses of the clonal lineage in carcinogenesis with the ability to call somatic mutations that are missed due to their low AFs [11, 16]. Nevertheless, these approaches still fail to address the fundamental limitation in the study design of explaining cancer evolution from CRC sample alone.

In this study, we used the cancer adjacent polyp as a snapshot of the origin of the CRC's clonal lineage to infer the pre-cancer time course. Numerous studies have reported that remnant features of primary tumors in their recurrent metastatic tumors, suggesting that the determination of whether the tumor will metastasize could possibly be present in the primary tumor before metastases have occurred [13, 15, 17].

We defined a set of criteria that is based on the information about SNVs and CNAs in both cancer and its matching polyp in order to classify each case into one of four MOEs. Other types of variations, such as transposons and indels, could be included in such classification. However, given that such variations are less frequent and that they are more difficult to detect, we did not consider them in this study. Our defined criteria are almost universal, as we were able to classify all but two cases into a distinct MOE with no ambiguity. Complications in classifying the two cases could stem from those cases not belonging to any of the established four MOEs, i.e., these two cases have undergone an extremely rare and not well-understood MOE such as CRC with polyclonal origin [11]. For every mode, we found at least one case corresponding to it. Approximately 70% of CRCs in our dataset evolved in a stepwise or a parallel fashion, although we should note that all cases are expected to exhibit features of parallel evolution, given the nature of our experiment, i.e., existence of matched polyp and CRC. We also found that the non-aggressive cases in which patients with stage II and III CRC survived their cancer exhibited all four stepwise, parallel, neutral, or eruptive MOE. At the same time patients with aggressive CRC, presenting either with metastatic disease or later developing recurrent disease, predominantly exhibit a stepwise and parallel MOE. This implies that MOE may not be the only determinant of clinical aggressiveness.

Our data on the relationship between the MOE and the somatic substitution mutations show that the patterns in the somatic substitutions were more significantly influenced by the underlying mutational mechanisms than by their MOEs. It also indicates that several different mutational mechanisms can lead to the same MOE and two different MOEs can have similar mutational mechanisms. Similarly, CNA patterns did not directly correlate with the MOE. This indicates that mutations in specific genes or pathways, and sequential order of deletions and duplications could determine the MOE of a CRC. While it is possible that factors other than the mutational mechanism—such as somatic variants in particular pathways, germline variants, and perhaps the microbiome—that can determine the MOE of a CRC, it is also possible that the relationship between MOE and mutational mechanism simply could not be found given our small sample size. Thus our finding warrants further study in understanding the relationship between the two in a larger sample set.

Overall, our study is the first to define specific criteria to link the MOEs to mutational characteristics in patient cases using cancer and its residual polyp of origin. Just as carcinogenesis models are relevant across different cancers, this MOE classification of CRC may apply to other cancer types with premalignant stages including the precancerous stages of pancreatic cancer known as pancreatic intra-epithelial neoplasia (PanIN) and ductal hyperplasia that precedes breast cancer [18, 19]. Use of MOE in the study of neoplastic transformation promises to provide additional insight into the genomic landscape of cancer as our classification signifies a characterization of cancer that is vastly different from the conventional genomic profiling using somatic SNVs and CNAs. While our criteria for MOE classification are mostly qualitative, development of a statistical approach to quantify the probability of being any of the MOEs is thus warranted to scale MOE classification to more cancers and cases.

Combining single cell analysis and intra-tumoral/intra-polyp heterogeneity approaches focusing on the more precise tracking of cancer evolution from the early, perhaps polyp-specific events initiating neoplastic transformation, will most likely provide additional insights into the details of malignant transformation in CRC. This insight, along with the relevance of CRC MOE modeling in other cancer types, may better our understanding of carcinogenesis in order to improve prognostication and to develop treatments targeted at the most relevant molecular events that drive both malignant transformation and ultimately progression.

## MATERIALS AND METHODS

### Patient sample characteristics

All tissues were collected from patients consented to the IRB approved Biobank for Gastrointestinal Health Research [BGHR] (IRB 622-00, PI LA Boardman) at Mayo Clinic between 2000–2016. 1 cm^2^ portions of surgically or endoscopically resected cancers from patients with CRC RPO+ were snap frozen in liquid nitrogen and maintained long term at −80°C. All of the CAP polyps were sessile and ranged in size from 2 to 6 cm. Matched normal colonic epithelium were collected at least 8cm away from the polyp/tumor margin. This study did not include subjects with family history of FAP or Lynch syndrome and any other hereditary CRC or inflammatory bowel disease.

### Tissue preparation and whole genome sequencing

An H and E slide circled by a pathologist to enrich for distant normal colon epithelium a minimum of 8 cm away from the polyp or tumor edge, polyp and cancer tissues was used a guide slide for macrodissection of these two tissue compartments. DNA from peripheral blood leukocytes (PBL) from the patients was obtained on a subset of these patients. DNA was extracted using the PureGene method and was quantified with appropriate kits on the Qubit Fluorometer. Samples were sequenced at the Broad Institute on the Illumina HiSeq X instruments producing 150 base pair, paired-end reads to meet a goal of at least 30× mean coverage. All data from a particular sample was aggregated into a single BAM file using the Picard Tools (https://broadinstitute.github.io/picard/).

### Mutation frequency detection

Four different somatic variant callers were used to identify SNVs in the polyp and cancer against the matched normal tissue or PBL with default options: MuTect, SomaticSniper, Strelka, and VarScan [20–23]. We only took SNVs detected by at least three callers. Variant allele frequencies for those SNVs were calculated from sample BAM files for each patient using an in-house script. For functional annotations of the variants, we used Variant Effect Predictor (http://www.ensembl.org/Tools/VEP).

### Mutation spectra analysis

From the list of somatic SNVs called in cancer, we subtracted somatic SNVs called in polyp to ensure mutual exclusivity between the cancer and polyp SNVs. Cancer samples in the cases A04, A09, A11, A14, and A15 did not have enough cancer specific somatic SNVs and thus all somatic SNVs found in cancer were included. Each somatic SNV within a sample was categorized into the corresponding transversion or transition substitution mutation into one of the 96 tri-nucleotide possibilities. After normalization, correlation coefficient was calculated based on these vectors of 96 integers for each sample and UPGMA-based hierarchical clustering was performed on the samples based on this coefficient. All the statistical analyses were performed using R software. Heatmaps were generated using the ggplot() function in the R package, ggplot2. Hierarchical clustering was performed with the hclust() function with default parameters. Correlations coefficients were calculated using the cor() function with the Pearson method.

### Pathway from related genes

For each of the CAP and cancer, a list of genes with somatic SNVs was submitted to the Database for Annotation Visualization and Integrated Discovery (DAVID) for functional pathway analysis. To account for the background mutation rate, only the genes determined by the MutSig to be significantly mutated were used in the analysis (*p*-value < 0.05). The number of significantly mutated genes for CAP and cancer were determined to be 123 and 137 respectively. The results of the multiple hypothesis tests were corrected using Benjamini method (FDR < 0.05). No particular pathway categories were significantly affected.

### CNA analysis

We called CNAs in cancers and polyps using CNVnator using bin size of 200 bps [24]. The regions of deletion and duplication were then genotyped and only the regions with a copy number of >1.75 and <2.25 in normal samples were considered. To further filter the CNAs, only the regions with copy number difference greater than 0.2 with respect to normal tissue were chosen. A pairwise similarity metric M is based on Jaccard similarity coefficient was defined as follows:

$$M=\underset{per\;Chr}{\sum }\frac{\sum^{\text{​}}R_{dup,dup}+\;\sum^{\;}\;R_{del,del}}{\sum^{\text{​}}R^{1}{}_{dup}+\sum^{\text{​}}R^{1}{}_{del}+\sum^{\text{​}}R^{2}{}_{dup}+\sum^{\text{​}}R^{2}{}_{del}-(\sum^{\text{​}}R_{dup,dup}+\;\sum^{\;}\;R_{del,del})}$$

The similarity metric per chromosome is equal to the sum of regions R of duplications and deletions common in both samples over the union of regions of duplication and deletion for the samples. Because these are scores per chromosome, calculating the summation of these scores represents a similarity score M between a pair of samples across their entire genome. Chromosomes X and Y were excluded from calculations to ensure metric comparability across genders. For each tissue type (adenoma or carcinoma), chromosomes with significantly recurrent aneuploidies compared to others were determined by a Wilcoxon signed-rank test. With a pairwise similarity metric across all the samples, UPGMA-based hierarchical clustering was performed. Heatmaps were generated by dividing the genomic regions into segments of 50kb and using the ggplot() function in the R package, ggplot2. Hierarchical clustering was performed with the hclust() function with default parameters.

### Distribution of materials and data

Whole genome data (raw BAM files) supporting the conclusions of this article are available in the dbGaP repository, with Study Accession number: phs001384.v1.p1. The study report can be accessed at: https://www.ncbi.nlm.nih.gov/projects/gap/cgi-bin/study.cgi?study_id=phs001384.v1.p1.

## SUPPLEMENTARY MATERIALS FIGURES AND TABLES







## Abbreviations

AF: Allele Frequency
CAP: Cancer Adjacent Polyp
CNA: Copy Number Alteration
CRC: Colorectal Cancer
CRC RPO+: Colorectal cancer with residual polyp of origin
CRC RPO-: Colorectal cancer without residual polyp of origin
FAP: Familial Adenomatous Polyposis
GBM: Glioblastoma Multiforme
ITH: Intratumoral Heterogeneity
MOE: Mode of Evolution
MSI: Microsatellite Instability
MSS: Microsatellite Stable
SNV: Single Nucleotide Variant
