# Supplementary material for: Inferring modes of evolution from colorectal cancer with residual polyp of origin
Source: Oncotarget. 2017 Dec 26;9(6):6780–92. doi: 10.18632/oncotarget.23687 (PMC5805514; doi:10.18632/oncotarget.23687)
Supplement: Supplementary file 1 [file oncotarget-09-6780-s001.pdf]

Inferring modes of evolution from colorectal cancer with residual polyp of origin

SUPPLEMENTARY MATERIALS

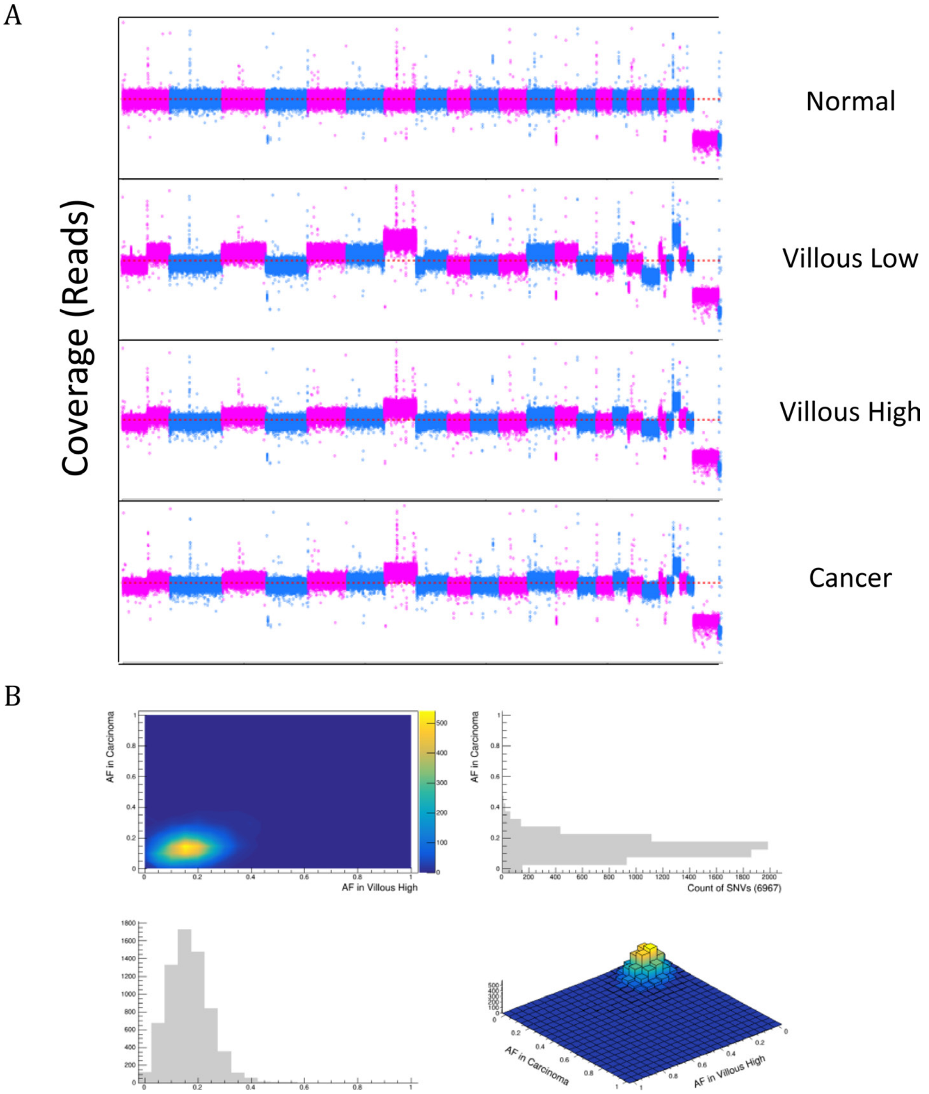

Supplementary Figure 1: Analysis summary for case A02, which is either a stepwise or parallel lineage. (**A**) Copy number profile across all chromosomes. Large aneuploidies are observed only at the cancer stage. (**B**) 2D allele frequency distribution is in agreement with the parallel scenario, but stepwise scenario is also possible (see Figure 1B). SNVs were found on TP53 at 0.567 AF (cancer); on FBXW7 at 0.556 AF (villous adenoma); on PIK3CA at 0.343 AF (villous adenoma); on BRAF at 0.455 (villous adenoma) and 0.431 AFs (cancer). 3D representation of the AF distributions of SNVs is shown on the bottom right. The height of the distributions, which shows the number of mutations, indicates large fraction of both the shared SNVs and private SNVs.

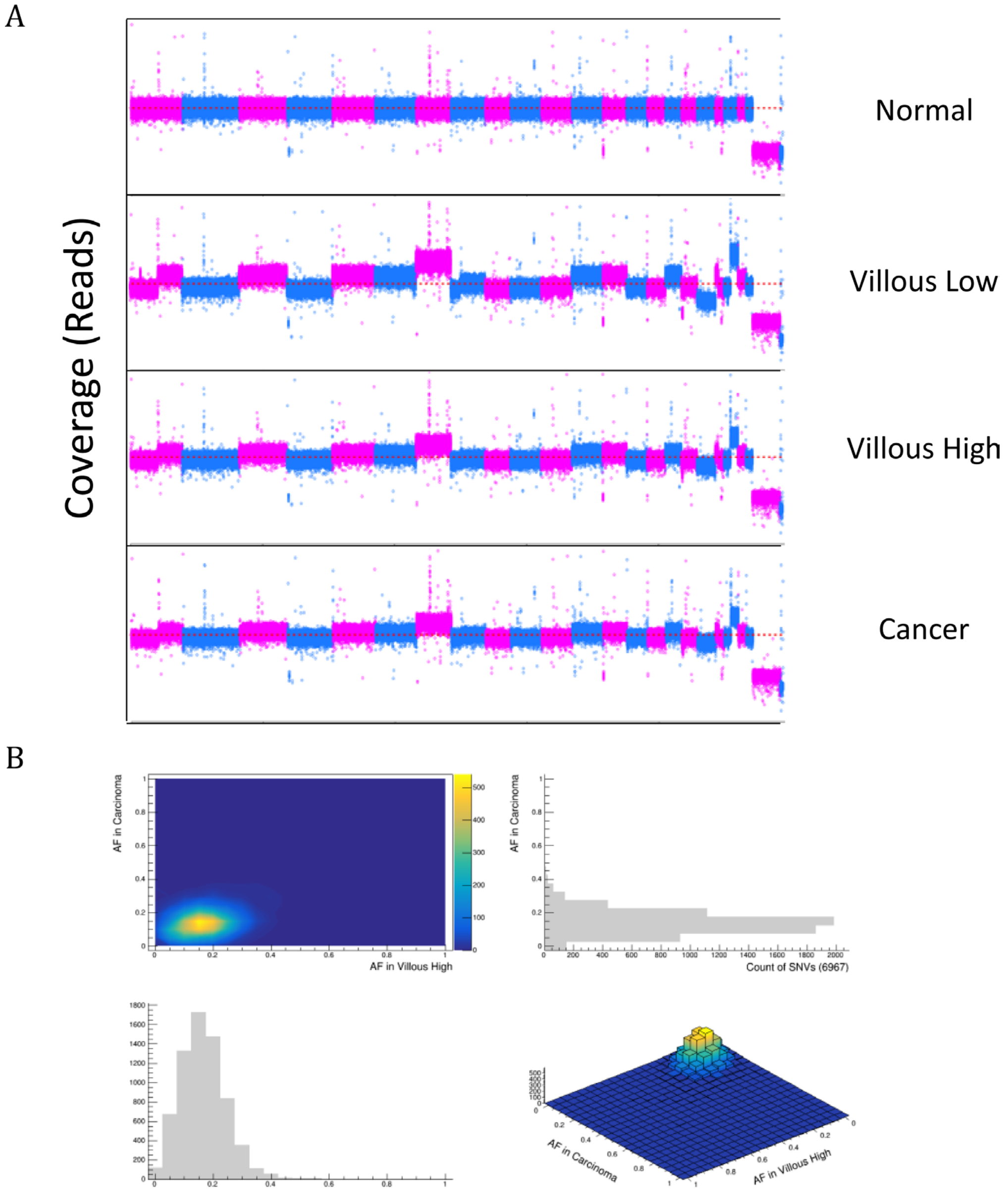

Supplementary Figure 2: Analysis summary for case A04, which is likely a neutral lineage. (**A**) Copy number profile across all chromosomes. Large aneuploidies are observed before the cancer stage. (**B**) 2D AF distribution is in agreement with the neutral lineage (see Figure 1B). SNVs were found on APC at 0.308 AF (tubular adenoma), 0.159 AF (villous adenoma), and 0.194 AF (cancer); on KRAS at 0.4 AF (tubular adenoma), 0.176 AF (villous adenoma), and 0.114 AF (cancer); on FBXW7 at 0.409 AF (tubular adenoma), 0.2 AF (villous adenoma), and 0.196 AF (cancer). 3D representation of the AF distributions of SNVs is shown on the bottom right. The height of the distributions, which shows the number of mutations, indicates that majority is shared SNVs.

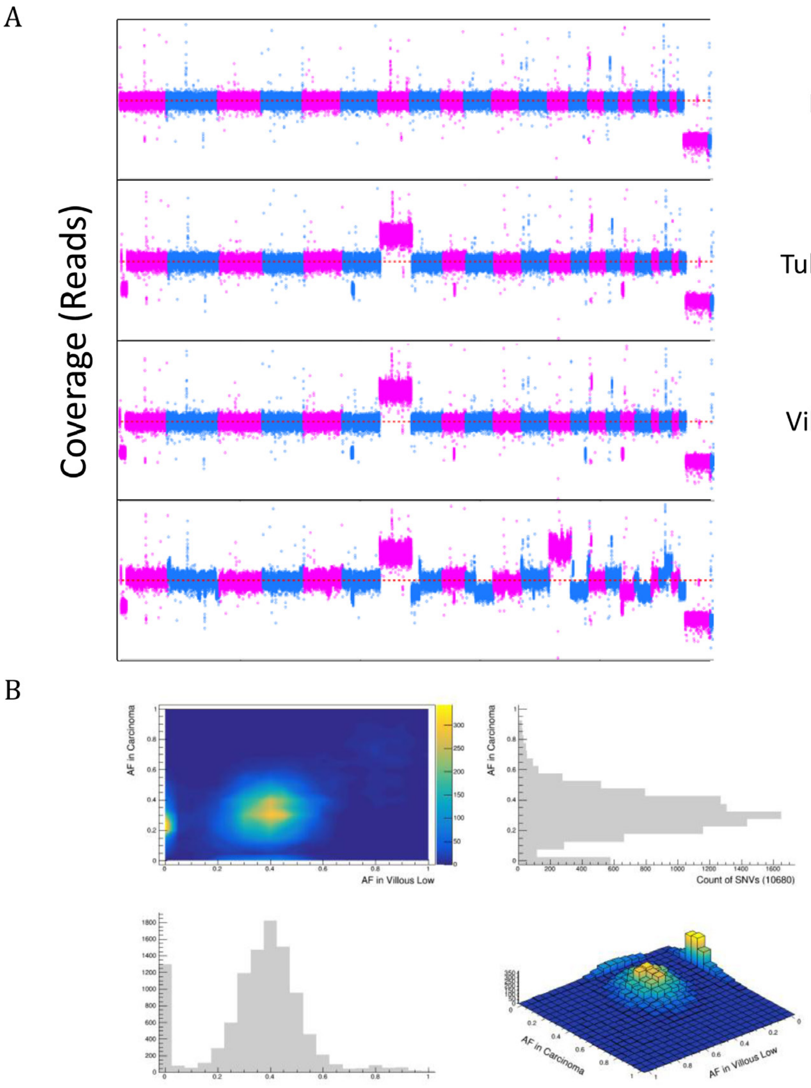

Supplementary Figure 3: Analysis summary for case A07, which is either a stepwise or parallel lineage. (**A**) Copy number profile across all chromosomes. The entire chromosome 7 is duplicated early in the progression, but large aneuploidies are not observed until later in the cancer stage. (**B**) 2D allele frequency distribution is in agreement with the parallel scenario, but stepwise scenario is also possible (see Figure 1B). SNVs were found on APC at 0.441 AF (tubular adenoma), 0.424 AF (villous adenoma), and 0.519 AF (cancer); on TP53 at 0.409 AF (cancer); on KRAS at 0.29 AF (tubular adenoma), 0.452 AF (villous adenoma), and 0.31 AF (cancer). 3D representation of the AF distributions of SNVs is shown on the bottom right. The height of the distributions, which shows the number of mutations, indicates large fraction of both the shared SNVs and private SNVs.

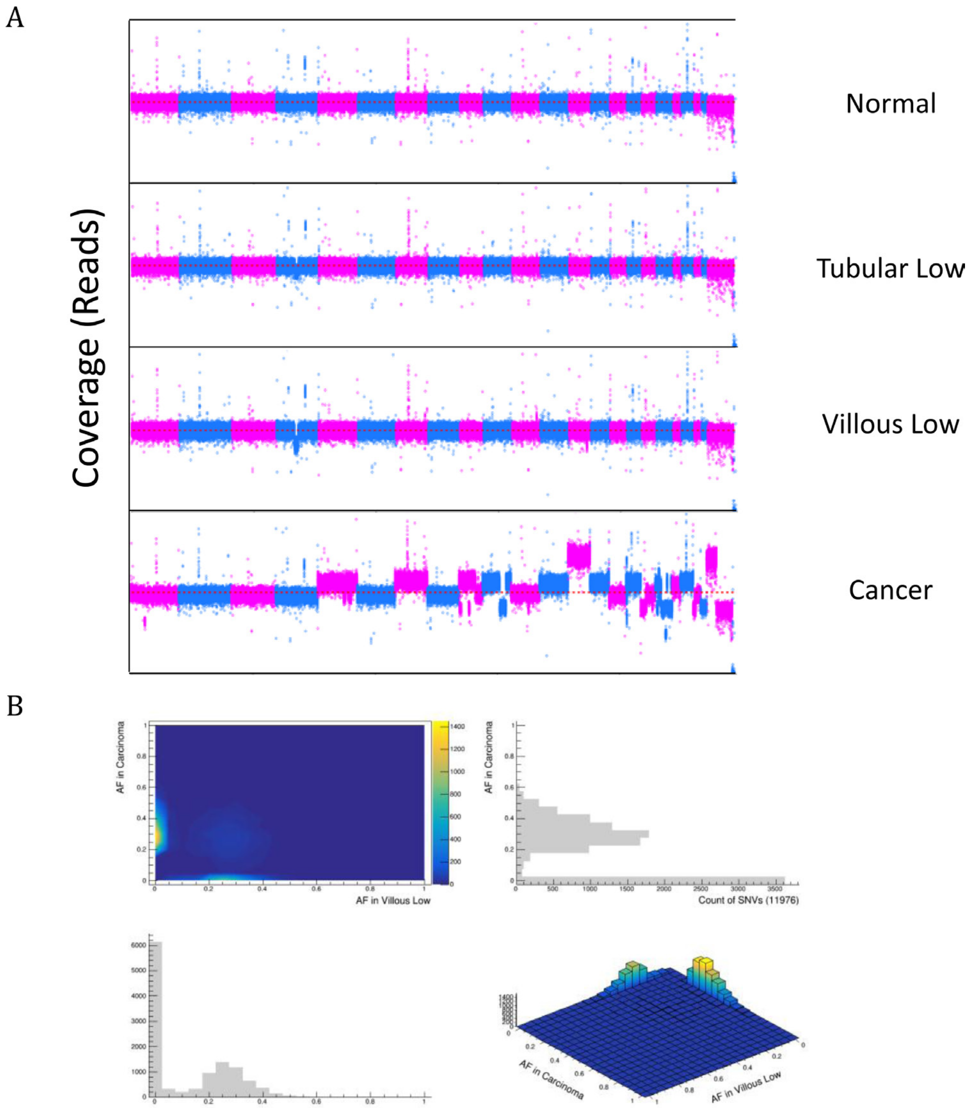

Supplementary Figure 4: Analysis summary for case A08, which is likely a parallel lineage. (**A**) Copy number profile across all chromosomes. The deletion on chromosome 4 progressively becomes larger until the cancer stage, at which it is no longer observed; it appears to be subclonal. Large aneuploidies are observed only at the cancer stage. (**B**) 2D AF distribution is in agreement with the parallel lineage (see Figure 1B). SNVs were found on APC at 0.156 AF (tubular adenoma), 0.154 AF (villous adenoma), and 0.2 AF (cancer); on TP53 at 0.448 AF (cancer); on KRAS at 0.25 AF (cancer); on PIK3CA at 0.294 AF (cancer). 3D representation of the AF distributions of SNVs is shown on the bottom right. The height of the distributions, which shows the number of mutations, indicates that majority is private SNVs.

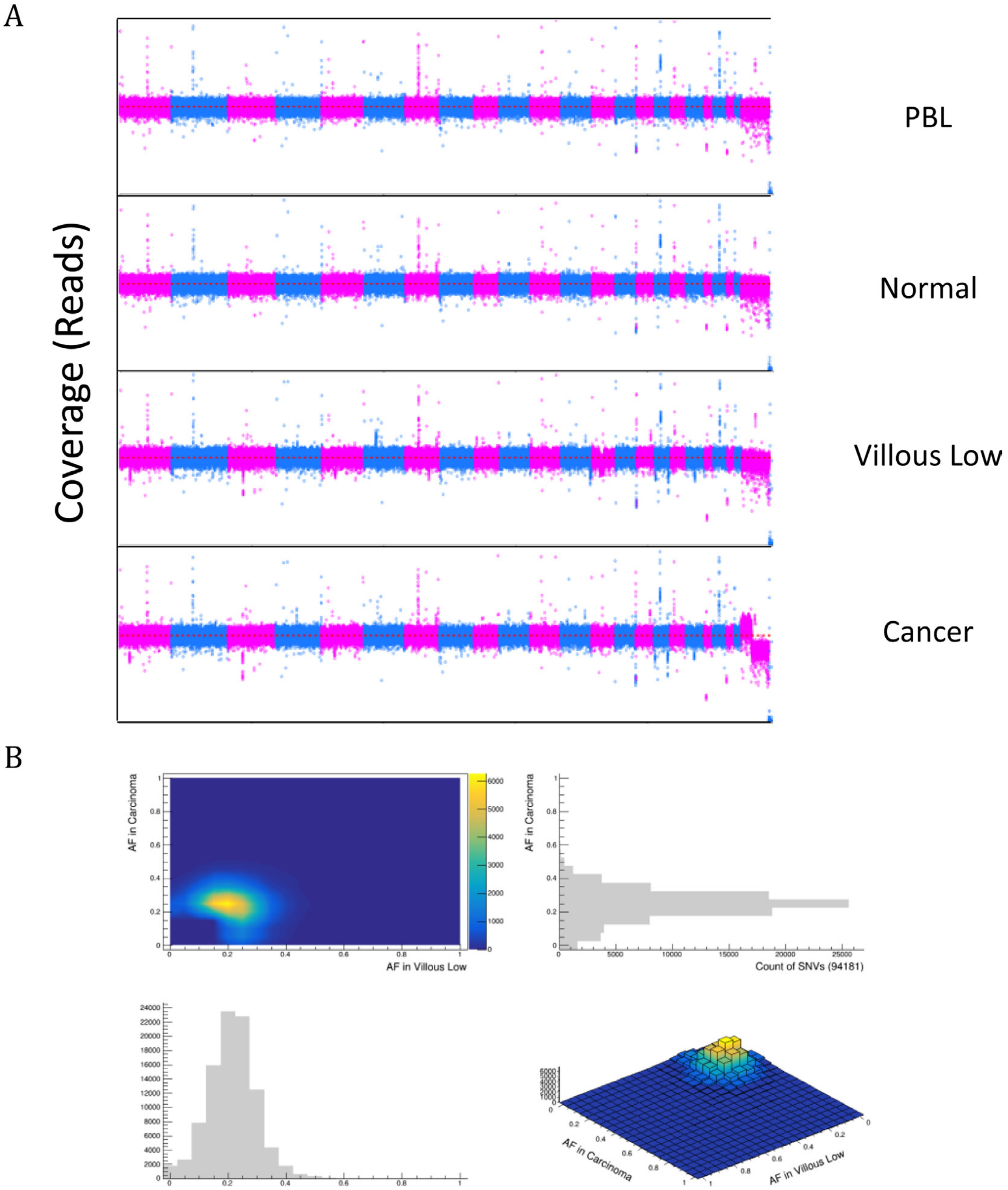

Supplementary Figure 5: Analysis summary for case A10, which is likely a neutral lineage. (**A**) Copy number profile across all chromosomes. No large aneuploidies are observed. (**B**) 2D allele frequency distribution is in agreement with the neutral lineage (see Figure 1B). SNVs were found on BRAF at 0.22 AF (villous adenoma) and 0.152 AF (cancer). 3D representation of the AF distributions of SNVs is shown on the bottom right. The height of the distributions, which shows the number of mutations, indicates that majority is shared SNVs.

A

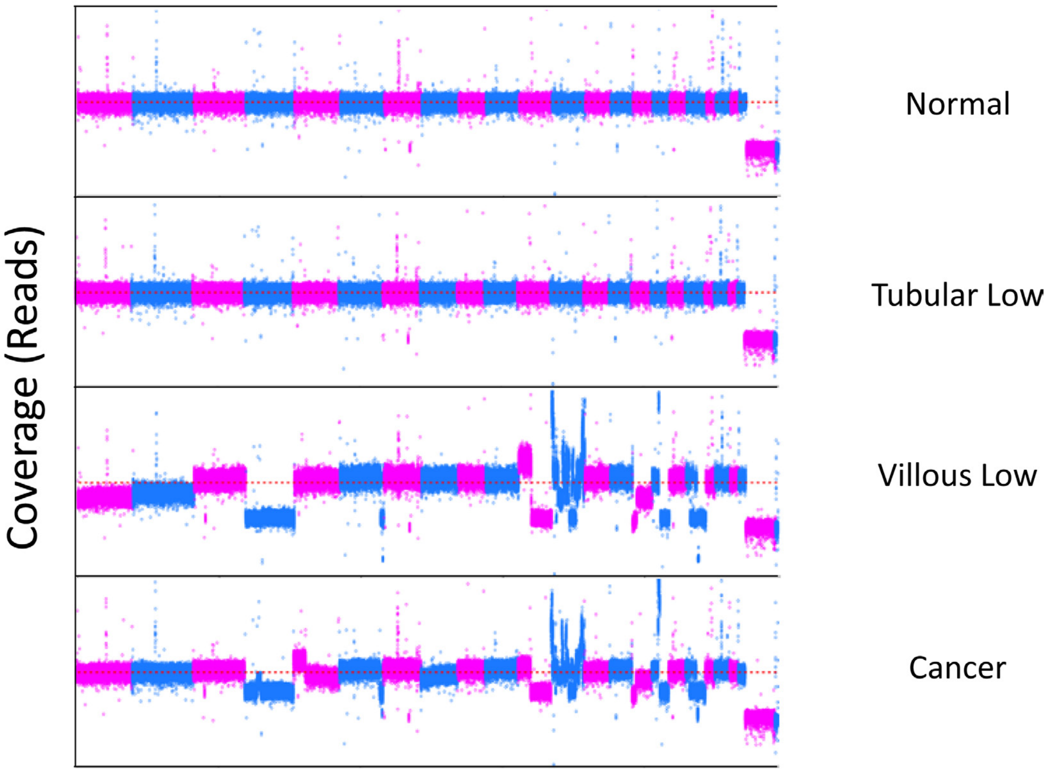

B

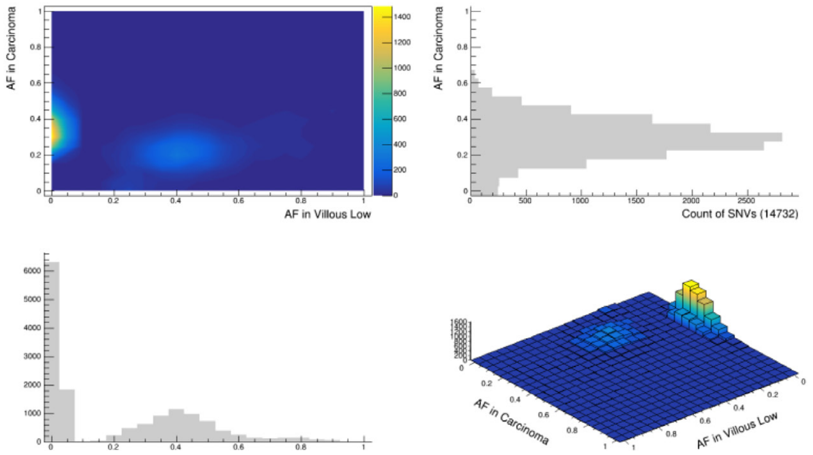

Supplementary Figure 6: Analysis summary for case A11, which is likely a stepwise lineage. (**A**) Copy number profile across all chromosomes. Large aneuploidies are observed before the cancer stage. (**B**) 2D allele frequency distribution is in agreement with the stepwise lineage (see Figure 1B). SNVs were found on TP53 at both 0.471 and 0.324 AFs (villous adenoma) as well as 0.167 and 0.152 AFs (cancer). 3D representation of the AF distributions of SNVs is shown on the bottom right. The height of the distributions, which shows the number of mutations, indicates large fraction of both the shared SNVs and private SNVs.

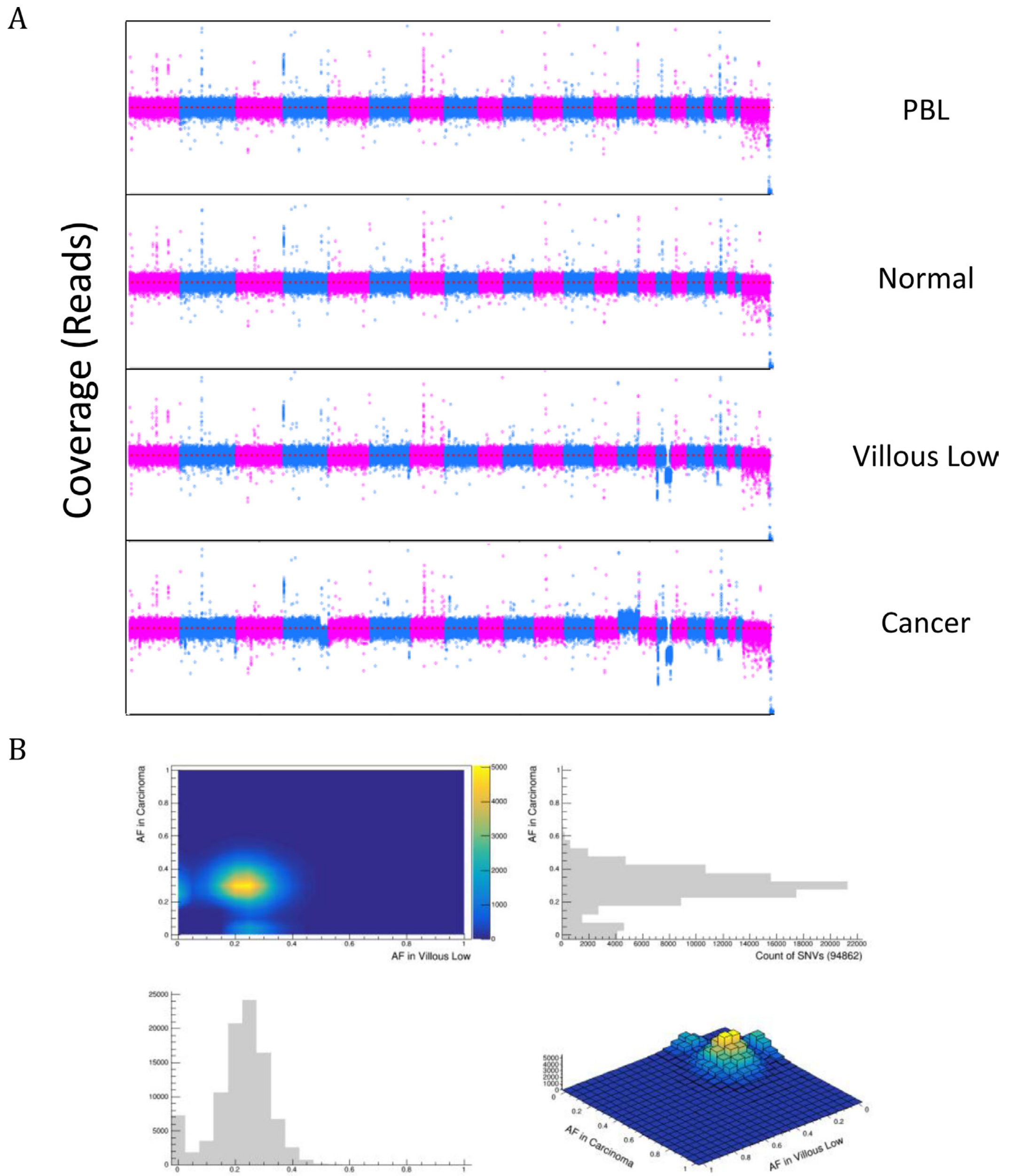

Supplementary Figure 7: Analysis summary for case A12, which is either a stepwise or parallel lineage. (**A**) Copy number profile across all chromosomes. No large aneuploidies are observed. (**B**) 2D allele frequency distribution is in agreement with the stepwise scenario, but stepwise scenario is also possible (see Figure 1B). SNVs were found on APC at 0.286 AF (villous adenoma) and 0.233 AF (cancer); on TP53 at 0.25 AF (cancer); on BRAF at 0.256 AF (villous adenoma) and 0.258 AF (cancer). 3D representation of the AF distributions of SNVs is shown on the bottom right. The height of the distributions, which shows the number of mutations, indicates large fraction of both the shared SNVs and private SNVs.

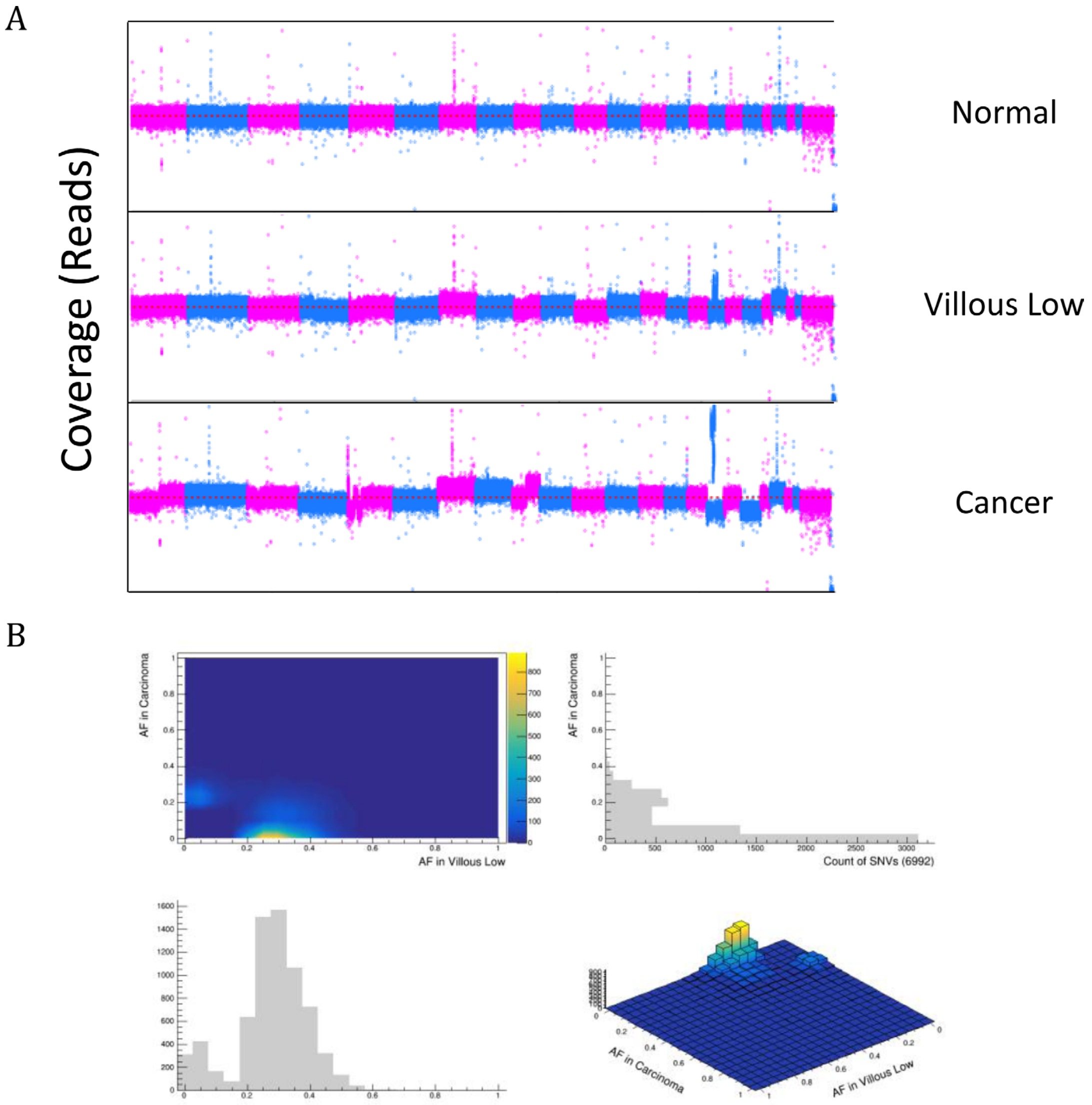

Supplementary Figure 8: Analysis summary for case A13, which is likely a parallel lineage. (**A**) Copy number profile across all chromosomes. Large aneuploidies are observed only at the cancer stage. (**B**) 2D allele frequency distribution does not follow any of the scenarios, but based on the number of mutations, it is likely to be a parallel lineage (see Figure 1B). SNVs were found on TP53 at 0.424 AF (villous adenoma) and 0.028 AF (cancer); on KRAS at 0.261 AF (villous adenoma). 3D representation of the AF distributions of SNVs is shown on the bottom right. The height of the distributions, which shows the number of mutations, indicates that majority is private SNVs.

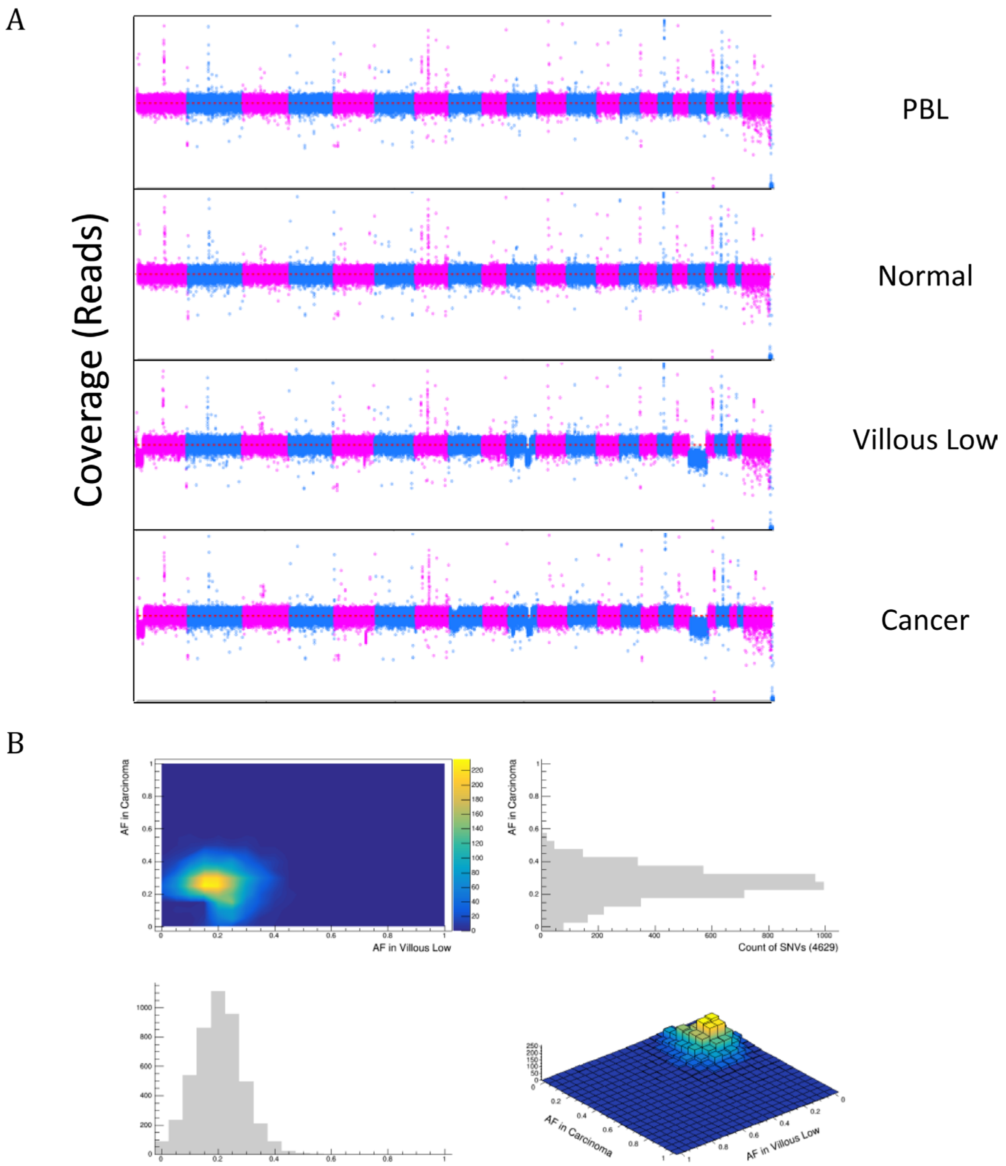

Supplementary Figure 9: Analysis summary for case A14, which is likely a neutral lineage. (**A**) Copy number profile across all chromosomes. No large aneuploidies are observed. (**B**) 2D AF distribution is in agreement with the neutral scenario (see Figure 1B). SNVs were found on APC at both 0.178 and 0.296 AFs (villous adenoma) as well as 0.333 and 0.357 AFs (cancer); on KRAS at 0.053 AF (villous adenoma) and 0.321 AF (cancer). 3D representation of the AF distributions of SNVs is shown on the bottom right. The height of the distributions, which shows the number of mutations, indicates that majority is shared SNVs.

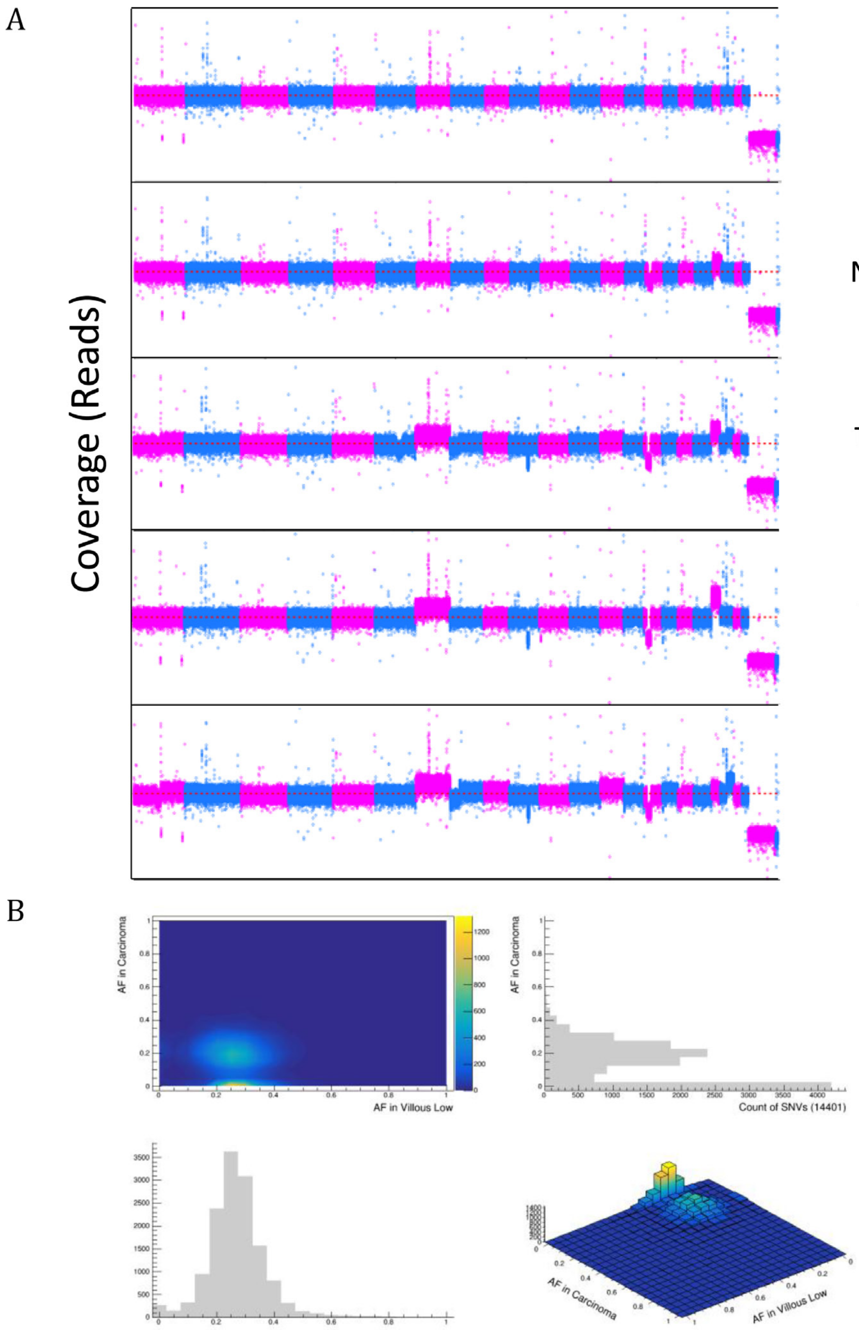

Supplementary Figure 10: Analysis summary for case A15, which is likely a parallel lineage. (**A**) Copy number profile across all chromosomes. No large aneuploidies are observed. (**B**) 2D AF distribution is in agreement with the parallel lineage (see Figure 1B). SNVs were found on APC at 0.071 AF (near normal), 0.275 AF (tubular adenoma), 0.31 AF (villous adenoma), and 0.207 AF (cancer); on KRAS at 0.125 AF (near normal), 0.294 AF (tubular adenoma), 0.481 AF (villous adenoma), and 0.267 AF (cancer). 3D representation of the AF distributions of SNVs is shown on the bottom right. The height of the distributions, which shows the number of mutations, indicates large fraction of polyp-specific SNVs and shared SNVs.

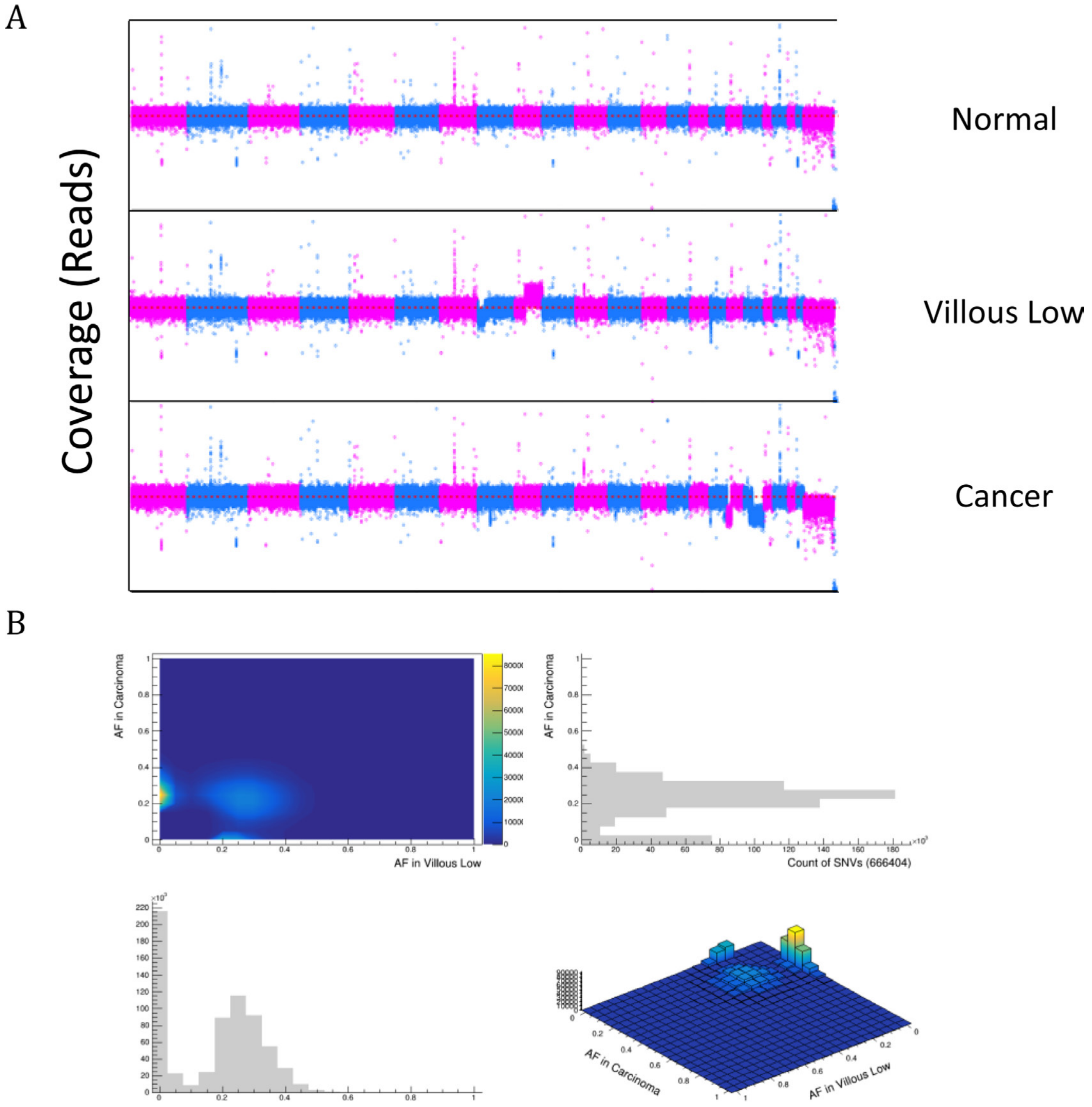

Supplementary Figure 11: Analysis summary for case A16, which is likely a stepwise lineage. (**A**) Copy number profile across all chromosomes. No large aneuploidies are observed. (**B**) 2D AF distribution is in agreement with the stepwise lineage (see Figure 1B). 3D representation of the AF distributions of SNVs is shown on the bottom right. The height of the distributions, which shows the number of mutations, indicates large fraction of both the shared SNVs and private SNVs.

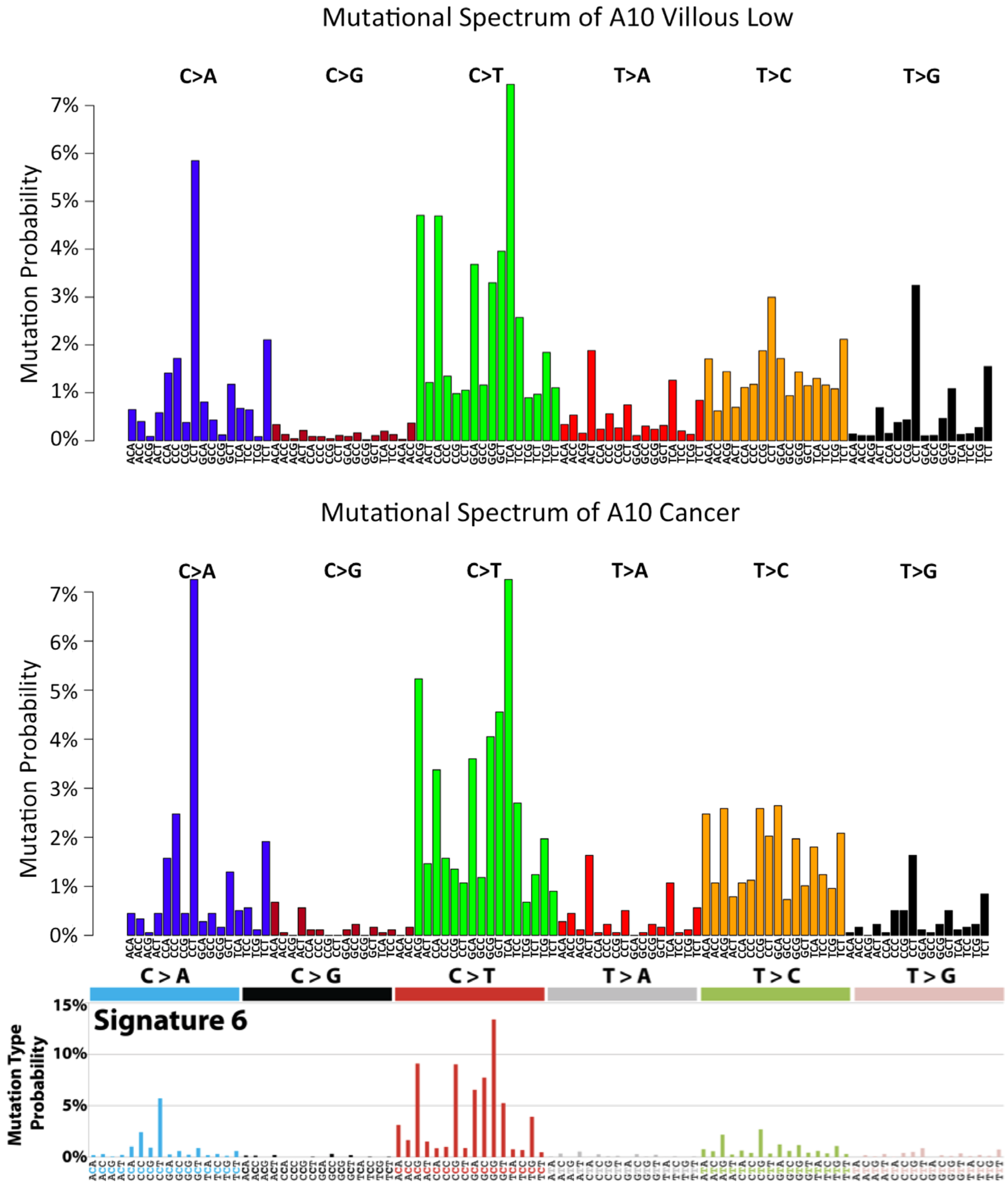

Supplementary Figure 12: Mutational spectra of the villous low and cancer in the case A10. The mutational spectra of the case A10 are compared to the mutation signature 6 from the COSMIC database [16]. The significant contributions from C>T and C>A substitutions clearly the resemble the case A10. Signature 6 is presumed to be associated with defective DNA mismatch repair found in MSI tumors (see http://cancer.sanger.ac.uk/cosmic/signatures).

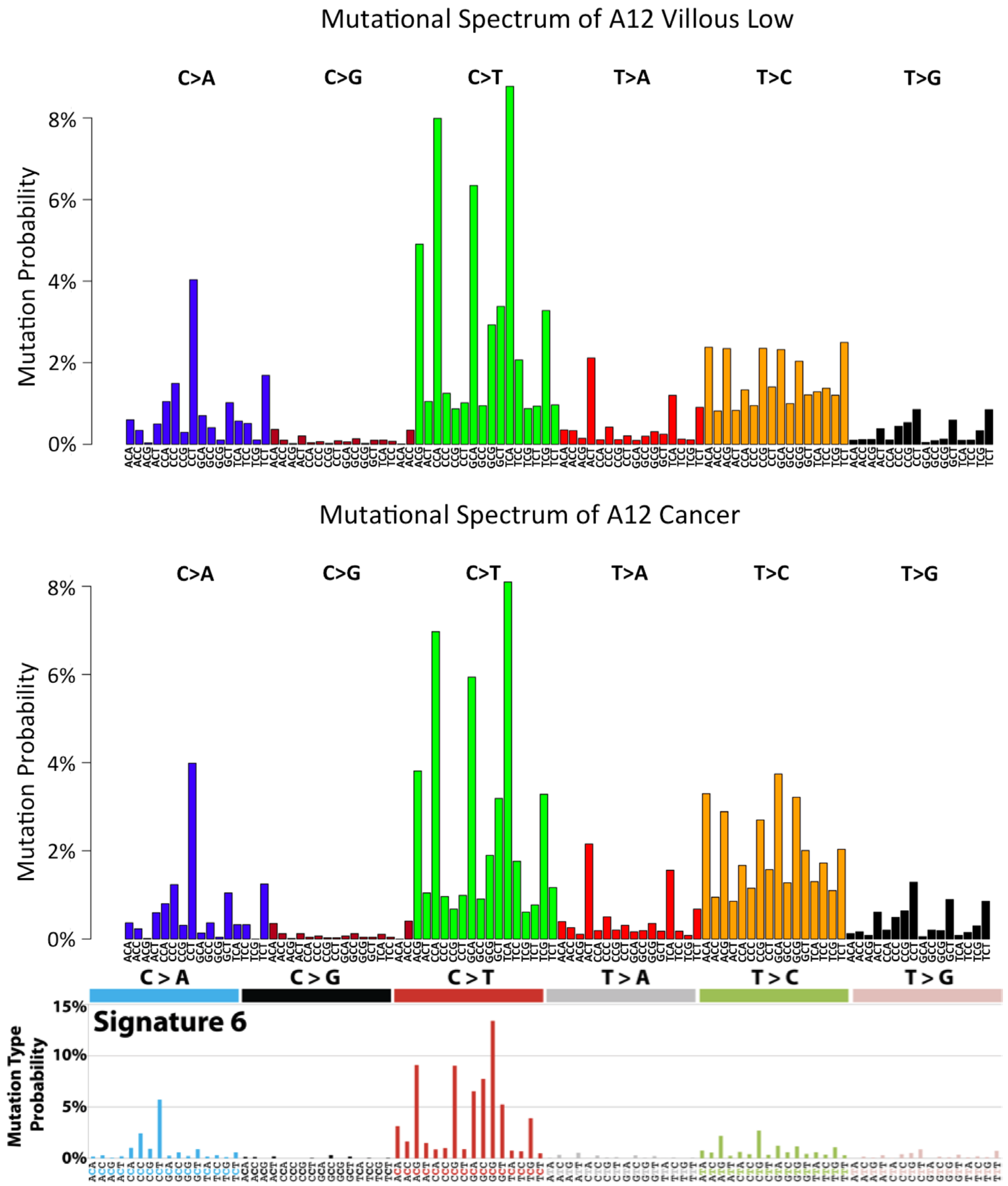

Supplementary Figure 13: Mutational spectra of the villous low and cancer in the case A12. The mutational spectra of the case A12 are compared to the mutation signature 6 from the COSMIC database [16]. The significant contributions from C>T and C>A substitutions clearly the resemble the case A12. Signature 6 is presumed to be associated with defective DNA mismatch repair found in MSI tumors (see http://cancer.sanger.ac.uk/cosmic/signatures).

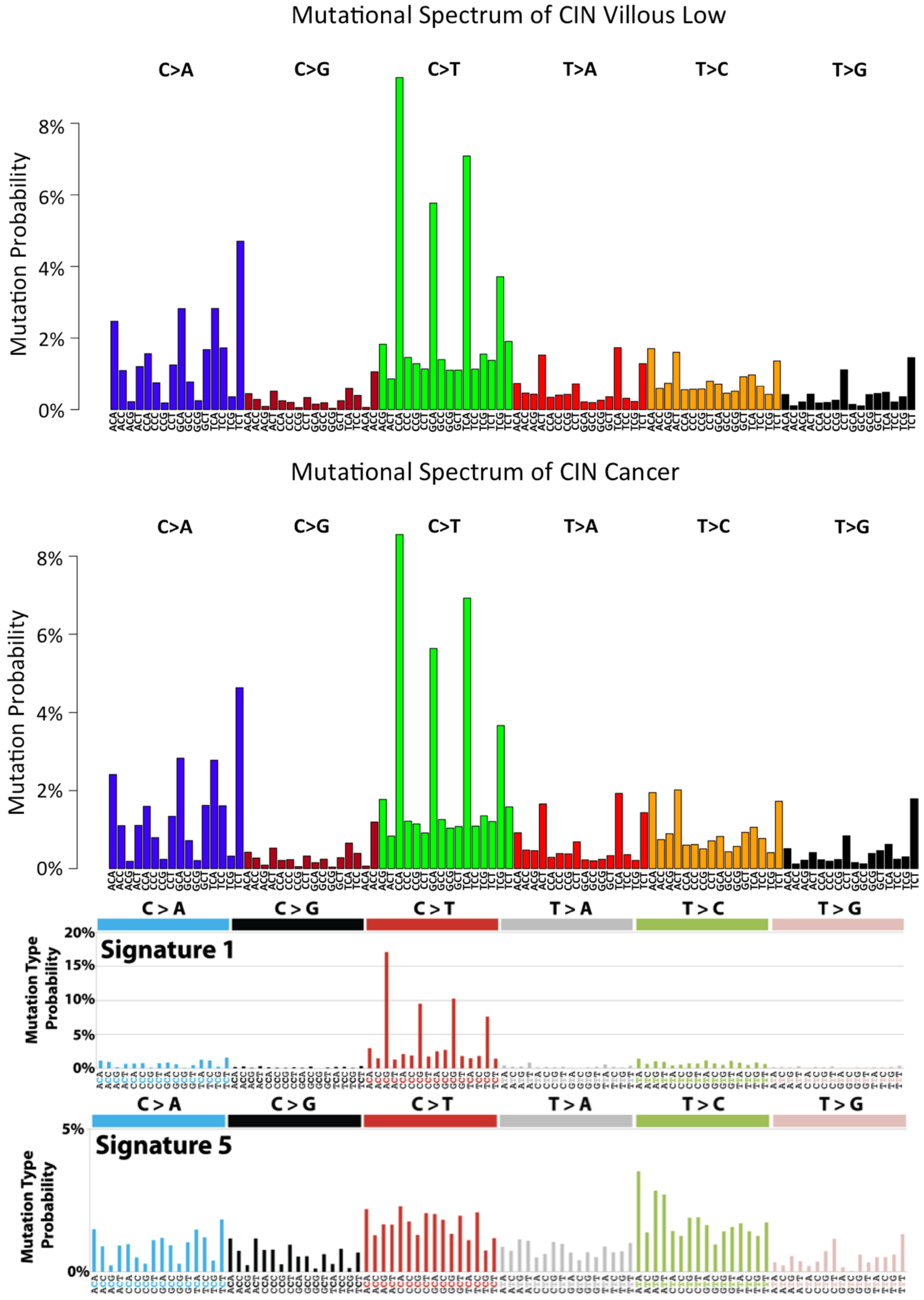

Supplementary Figure 14: Mutational spectra of the villous low and cancer in the CIN cases. The mutational spectra of the CIN cases are compared to the mutation signature 1 and 5 from the COSMIC database [16]. The significant contributions from C>A and C>T substitutions clearly the resemble the CIN cases. Signature 1 is presumed to be associated with sporadic deamination of 5-methylcytosine but signature 5 is not yet known to be associated with particular mechanisms (see http://cancer.sanger.ac.uk/cosmic/signatures).

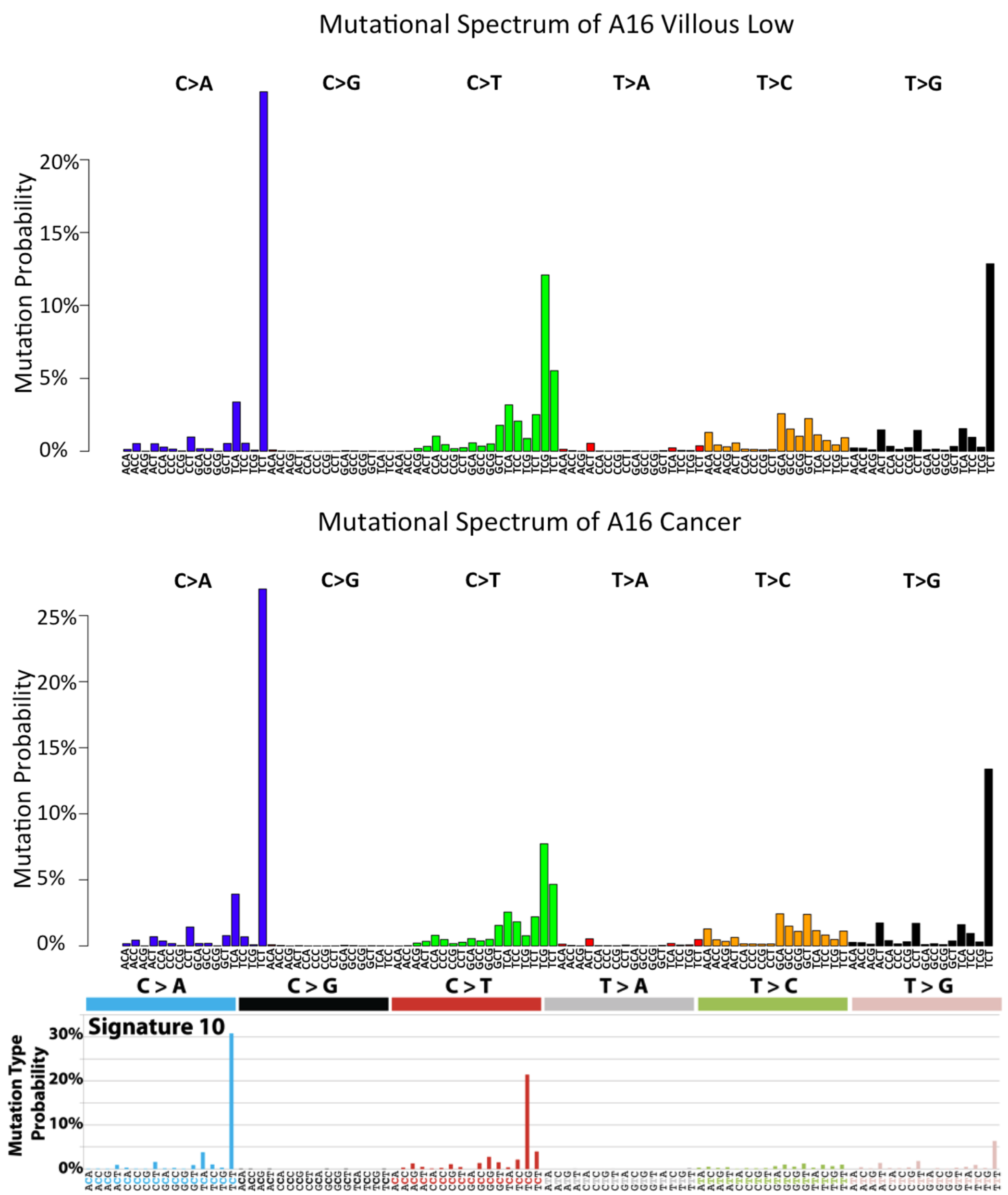

Supplementary Figure 15: Mutational spectra of the villous low and cancer in the case A16. The mutational spectra of the case A16 are compared to the mutation signature 10 from the COSMIC database [16]. The significant contributions from C>T and C>A substitutions clearly the resemble the case A16. Signature 10 is presumed to be associated with mutations in POLE gene (see http://cancer.sanger.ac.uk/cosmic/signatures).

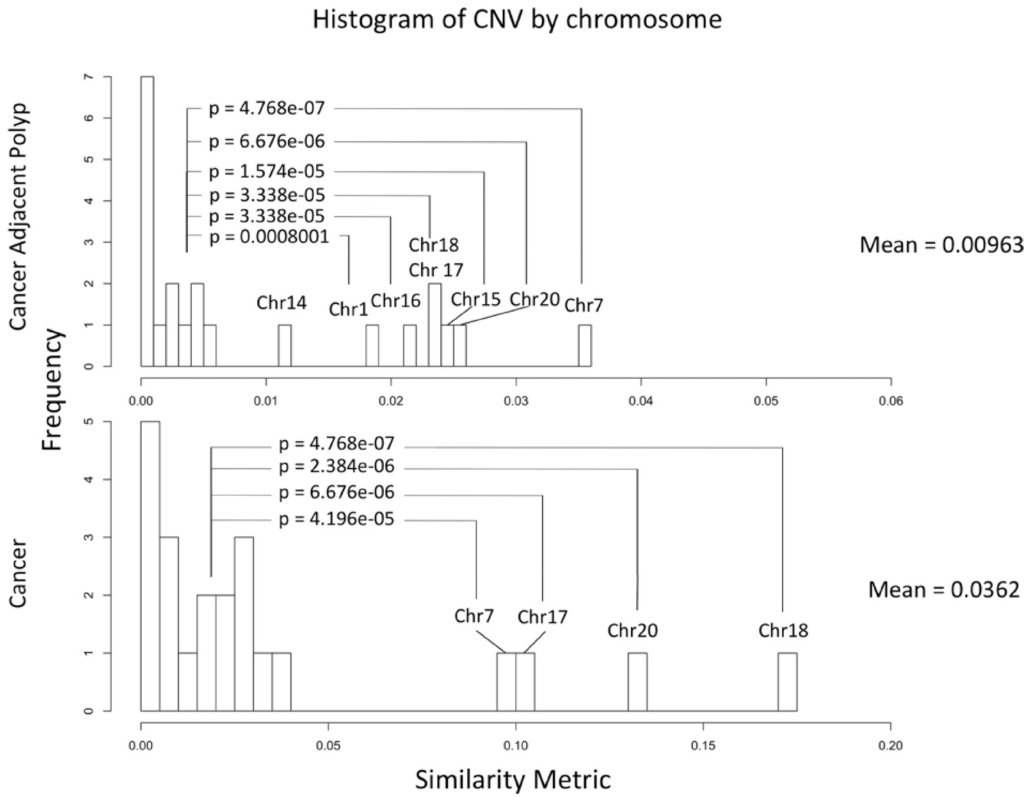

Supplementary Figure 16: Histogram of the similarity in the CNA per chromosomes for CAP and cancer. For CAPs, copy number changes in the chromosomes 7, 20, 15, 18, 17, 16, and 1 were significantly recurrent compared to the other chromosomes. For cancer samples, copy number changes in the chromosomes 18, 20, 17, and 7 were significantly more recurrent compared to the other chromosomes. Duplications in chromosome 7 and 20 were significantly recurrent in both CAP (*p*-value of 4.8×10^{-7} and 6.7×10^{-6}, respectively) and cancer (*p*-value of 4.2×10^{-5} and 2.4×10^{-6}, respectively). Deletions in chromosomes 17 and 18 were also significantly recurrent compared to other chromosomes in both CAP and cancer (*p*-values $< 3.4 \times 10^{-5}$).

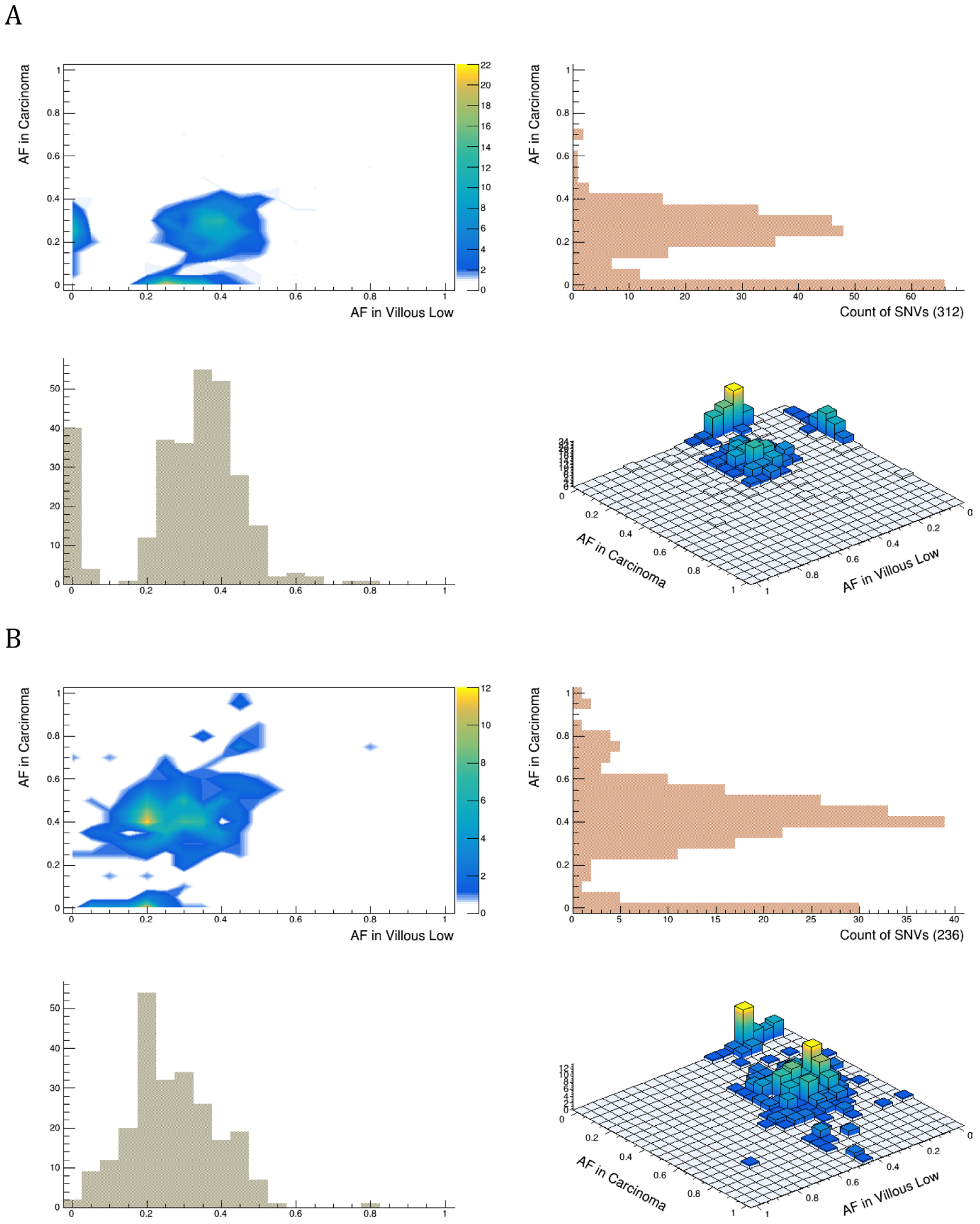

Supplementary Figure 17: Allele frequency distributions of SNVs in the coding regions only. (**A**) 2D and 3D AF distributions of SNVs in coding regions for case A03. General shape and the two gaps representing the stepwise evolution remain observable. (**B**) 2D and 3D AF distributions of SNVs in coding regions for case A09. General shape and the single gap in cancer representing the eruptive evolution remain observable.

Supplementary Table 1: Demographics of the 13 CRC patients. See Supplementary_Table_1

Supplementary Table 2: Pathological characterization, MOE assignment, number of SNVs, tumor purity, aneuploidy level, AF of selected driver mutations for all cases. See Supplementary_Table_2
